# Comparative transcriptomics of the model mushroom *Coprinopsis cinerea* reveals tissue-specific armories and a conserved circuitry for sexual development

**DOI:** 10.1186/1471-2164-15-492

**Published:** 2014-06-19

**Authors:** David Fernando Plaza, Chia-Wei Lin, Niels Sebastiaan Johannes van der Velden, Markus Aebi, Markus Künzler

**Affiliations:** Department of Biology, Institute of Microbiology, ETH Zürich, Zürich, Switzerland

**Keywords:** Fungal defense, Sexual development, *C. cinerea*, Primordia, RNA-seq, Comparative transcriptomics, Tissue-specific LC-MS proteome

## Abstract

**Background:**

It is well known that mushrooms produce defense proteins and secondary metabolites against predators and competitors; however, less is known about the correlation between the tissue-specific expression and the target organism (antagonist) specificity of these molecules. In addition, conserved transcriptional circuitries involved in developing sexual organs in fungi are not characterized, despite the growing number of gene expression datasets available from reproductive and vegetative tissue. The aims of this study were: first, to evaluate the tissue specificity of defense gene expression in the model mushroom *Coprinopsis cinerea* and, second, to assess the degree of conservation in transcriptional regulation during sexual development in basidiomycetes.

**Results:**

In order to characterize the regulation in the expression of defense loci and the transcriptional circuitries controlling sexual reproduction in basidiomycetes, we sequenced the poly (A)-positive transcriptome of stage 1 primordia and vegetative mycelium of *C. cinerea* A43mutB43mut. Our data show that many genes encoding predicted and already characterized defense proteins are differentially expressed in these tissues. The predicted specificity of these proteins with regard to target organisms suggests that their expression pattern correlates with the type of antagonists these tissues are confronted with. Accordingly, we show that the stage 1 primordium-specific protein CC1G_11805 is toxic to insects and nematodes. Comparison of our data to analogous data from *Laccaria bicolor* and *Schizophyllum commune* revealed that the transcriptional regulation of nearly 70 loci is conserved and probably subjected to stabilizing selection. A Velvet domain-containing protein was found to be up-regulated in all three fungi, providing preliminary evidence of a possible role of the Velvet protein family in sexual development of basidiomycetes. The PBS-soluble proteome of *C. cinerea* primordia and mycelium was analyzed by shotgun LC-MS. This proteome data confirmed the presence of intracellular defense proteins in primordia.

**Conclusions:**

This study shows that the exposure of different tissues in fungi to different types of antagonists shapes the expression pattern of defense loci in a tissue-specific manner. Furthermore, we identify a transcriptional circuitry conserved among basidiomycetes during fruiting body formation that involves, amongst other transcription factors, the up-regulation of a Velvet domain-containing protein.

**Electronic supplementary material:**

The online version of this article (doi:10.1186/1471-2164-15-492) contains supplementary material, which is available to authorized users.

## Background

The last eukaryotic common ancestor (LECA) was facultatively sexual and evolved nearly 1.5 billion years ago in the Proterozoic eon [1]. Sexual reproduction (SR) shares common features across the eukaryotic lineage such as ploidy changes, meiotic recombination and cell-cell recognition between gametes followed by cellular fusion and zygote formation [2]. An increase in the genetic diversity of the population, making it more adaptable to changing environmental conditions, as well as the dilution of deleterious mutations out of the gene pool are the most remarkable evolutionary innovations achieved by SR [3]. Despite all these clear benefits, sex is energetically expensive and entails a higher chance of genetic and organelle conflicts [2].

During sexual reproduction, fungi undergo dramatic morphological changes driven by environmental conditions such as light, nutrient availability and grazing by predators [4]. In basidiomycetes, mushroom development starts with intense localized hyphal branching leading to the formation of hyphal knots. These branching hyphae further aggregate to form 1–2 mm secondary nodules where cell differentiation leads to the establishment of bipolar primordia containing all the tissues observed in the mature fruiting body [5]. As a last step, primordia develop to mature fruiting bodies mainly by cellular expansion [6]. Due to their hyphal density, primordia and fruiting bodies are attractive to predators including mollusks, arthropods and nematodes [4].

*Coprinopsis cinerea* has been used as a model basidiomycete since the mid-1950s [7] due to its saprobic lifestyle, its rapid growth and the feasibility of producing fruiting bodies under defined laboratory conditions [6]. In nature, *C. cinerea* grows on horse dung [6], a eutrophic substrate rich in competing microorganisms, such as Firmicutes, Bacteroidetes and Proteobacteria [8]. The recent sequencing of the *C. cinerea* genome [9] allows the study of this organism gene expression on transcriptome and proteome level at different developmental stages or under a variety of environmental settings.

Morphological changes and environmental signals during fruiting body formation in *C. cinerea* are well described [6]; nonetheless, comparably little is known about the molecular machinery driving sexual reproduction processes in this basidiomycete. Recently, mutations blocking fruiting body development at different stages or altering mushroom morphology were identified [10–15]. In addition, *dst2* and *dst1*, encoding a blue-light photoreceptor and a flavin adenine dinucleotide-binding protein, were shown to play a role in blue light sensing. In agreement with previous experiments, strains defective in these two proteins were unable to form fruiting bodies, showing that blue light is an essential environmental trigger of mushroom development [16]. Most recently, *C. cinerea* strains carrying mutations in the putative component of the SWI/SNF chromatin remodeling complex *snf5* (CC1G_15539) were shown to be defective in fruiting initiation, suggesting that epigenetic reprogramming of loci occurs during fruiting body formation [17].

Aerial fruiting bodies are an attractive prey for predators and thus are protected by a battery of defense molecules (toxins) including proteins [18–21], peptides [22] and secondary metabolites [23–25]. Some of these toxins are known to be specifically produced in the fruiting body and not in the vegetative mycelium [26, 27]. For instance, cytoplasmic lectins showing a broad range of non-self-carbohydrate specificities, also referred to as fruiting body lectins due to their specific expression pattern, have been shown to exert toxicity to nematodes, insect larvae and amoeba [18]. Protein-mediated inhibition of serine proteases [20], proteolytic degradation of predator-derived proteins [28, 29] and sequestration of biotin [19] are other strategies of basidiomycetous fruiting bodies to dissuade predators. Vegetative mycelium, in contrast, digests extracellular carbon macromolecules in the growth substrate, such as cellulose and lignin, into smaller degradation products which are absorbed by the growing hyphae. At the same time, these smaller molecules become available for competing bacteria that profit from the fungal enzymatic machinery [30]. As a response to bacterial competitors, fungi have evolved secreted antimicrobial proteins. Mygind and colleagues presented recent evidence that vegetative mycelia of fungi secrete cysteine-stabilized antibacterial peptides which play a role in the arms race with competing bacteria [31].

Using RNA-seq in *C. cinerea*, we show evidence suggesting a role of the Velvet protein regulon in sexual development of basidiomycetes. Our data supports the existence of a conserved transcriptional circuitry in basidiomycetes fruiting bodies consisting of at least 60 orthologous genes probably involved in mushroom development and function. In addition, our data reveals the existence of two different sets of fungal defense proteins in vegetative and sexual organs matching the type of competitors and predators by which these structures are challenged in nature. The transcriptome data is supported by the first partial shotgun mass spectrometry catalog of proteins present in *C. cinerea* stage 1 primordia (S1P) and vegetative mycelium (VM).

## Results

### Differential gene expression during fruiting body development in *C. cinerea*

**Figure 1:**
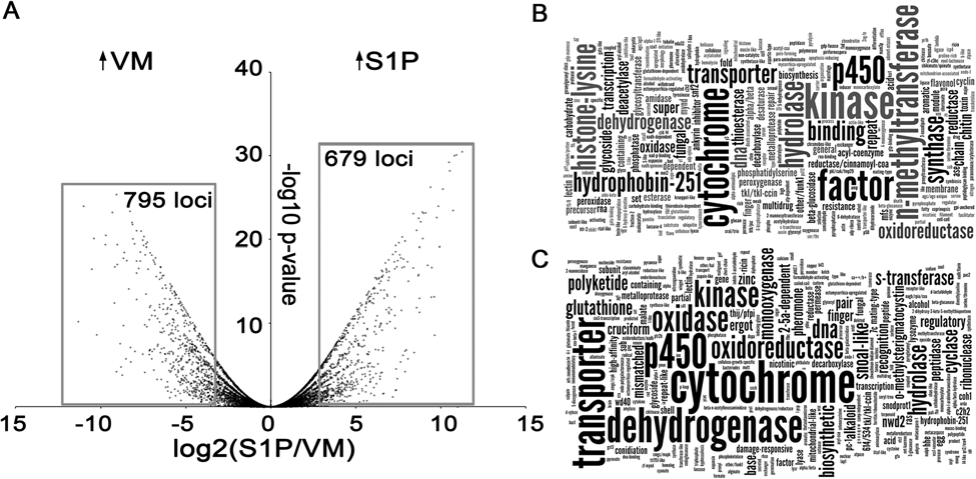
**Differential gene expression during early fruiting body development in** ***C. cinerea*** **. (A)** Volcano plot illustrating the relative expression (log2(S1P/VM)) and statistical significance of the 13342 loci in the genome of *C. cinerea* between VM and S1P. Fisher’s exact test -log10 p-value ≥ 1.3 (p-value ≤ 0.05) and log2(S1P/VM) +/- 3 (8 fold change) were used as thresholds of differential gene expression. Grey boxes comprise genes significantly up-regulated in S1P (right) or VM (left). Tag clouds showing enriched PSI-BLAST functional annotation terms from up-regulated loci in S1P **(B)** and VM **(C)** were produced in Wordle (© IBM Corporation) after removing frequently appearing tags.

Four different cDNA libraries were sequenced with a final 7.32 Gb mapped data output. Approximately 95% of the open reading frames (ORFs) in the genome of *C. cinerea* A43mutB43mut (AB) were transcribed using five reads/ORF as the minimal threshold for a locus to be considered as expressed (Table 1). Differential gene expression at the RNA level between S1P and VM was examined (Additional file 1: Table S1). Eleven percent of the annotated ORFs in the *C. cinerea* genome were found to be differentially expressed, 795 (6%) and 679 loci (5%) in VM and S1P, respectively, using fold change 8 and Fisher’s exact test p-value ≤ 0.05 as thresholds (Figure 1, Additional file 2: Table S2). The number of differentially transcribed loci in these two developmental stages increased to 2522 in VM and 3209 in S1P when a fold change threshold of 2 was set, corresponding to approximately 45% annotated ORFs in the genome of *C. cinerea*.

**Table 1:** **General features of the** ***C. cinerea*** **S1P and VM transcriptomes**

	Library size (reads)		
	Biological replicate 1	Biological replicate 2	Expression (%)*
**S1P**	26243754	25653679	95.55
**VM**	29374279	23269631	95.13
	**Average SOLiD read size (b)**	70	
	**Sequenced material (Gb)**	7.32	

*Mean percentage of annotated ORFs detected to be expressed using a minimal threshold 5 reads/ORF.

To validate these data, qRT-PCR of four selected loci (CC1G_10318, CC1G_09480, CC1G_05299 and CC1G_11805) was performed. Although S1P/VM expression ratios did not exactly match those observed by RNA-seq, qRT-PCR results showed the same trend of differential gene expression for these loci during *C. cinerea* development as observed by SOLiD RNA-seq (Additional file 3: Figure S1). In addition, we verified that the expression values of reported housekeeping loci commonly used in qRT-PCR normalization were in a range indicative of constitutive expression (-2 ≤ log2(S1P/VM) ≤ 2) (Additional file 4: Table S3).

Enrichment of PSI-BLAST-derived functional annotation terms was visualized using Wordle (© IBM Corporation) [32] after excluding frequent non-informative terms. By far, the most commonly assigned annotation term was “hypothetical protein” in S1P (275 loci) and VM (426 loci); nonetheless, there was a significant enrichment of N-methyltransferase (histone-lysine N-methyltransferase) tags in S1P (7 loci). Enrichment of functional annotation terms also shows that different sets of cytochromes, kinases, dehydrogenases, transporters and hydrophobins are specifically expressed in vegetative mycelium or young fruiting bodies (Figure 1). Functional annotation clustering using DAVID Bioinformatics Resources 6.7 [33] confirmed the enrichment of protein methyltransferases and hydrophobins in S1P with 11 and 6 fold enrichment compared to the occurrence of these functional categories in the genome of *C. cinerea* (Additional file 5: Table S4).

### Tissue-specific expression of *C. cinerea*defense proteins

Several cytoplasmic defense lectins and protease inhibitors such as *C. cinerea* lectin 2 (CCL2, CC1G_11781) [34], *Coprinopsis* galectin 1 (CGL1, CC1G_05003) [35] and two paralogous serine protease inhibitors from *Coprinopsis* (Cospin; CC1G_09479 and CC1G_09480) [20] were found to be highly up-regulated in *C. cinerea* S1P (Table 2). Out of these previously characterized genes coding for proteins with nematotoxic and insecticidal activity, Cospin and CCL2 were found to be among the top 50 most highly transcribed and differentially expressed loci in S1P. Among these, two loci, CC1G_10318 and CC1G_11805, encoding homologous proteins with a predicted aerolysin/ETX pore-forming domain as well as loci encoding proteins with suspected antibacterial and antifungal function including two peptidoglycan binding proteins, the toxin component of a bacterial toxin-antitoxin system and a Thaumatin-like protein were found. In contrast to S1P-specific defense proteins, defense proteins specifically up-regulated in VM were mainly secreted. These proteins included three putative lysozymes, several proteins containing a CFEM domain (PF05730) whose structure resembles cysteine-stabilized antibacterial peptides, and two representatives of the cerato-platanin family of secreted proteins (Table 3). Latter protein family is expanded in basidiomycetes and has recently been implicated in interactions of dikaryotic fungi with other organisms [36, 37]. During sexual development on herbivore dung, *C. cinerea* is exposed to a succession of antagonists (predators and competitors) colonizing this substrate. The differential expression of cytoplasmic and secreted defense proteins in S1P and VM, respectively, might reflect the prevalent types of antagonists with which these tissues of *C. cinerea* are confronted.

**Table 2:** **S1P-specific defense loci**

Locus	Fold S1P/VM	Functional annotation	*p-value	†SignalP	‡TMHMM
CC1G_09480	2426	Cospin1	1.26E-305	N	N
CC1G_11781	1939	CCL2	4.64E-302	N	N
CC1G_09479	692	Cospin2	2.38E-132	N	N
CC1G_12219	237	Related to Velvet A protein	6.42E-43	N	N
CC1G_07937	83	Ricin B-fold protein	1.08E-148	Y	N
CC1G_11778	69	CCL1	7.80E-137	N	N
CC1G_10318	68	Pore-forming protein	8.08E-91	N	N
CC1G_07956	66	Peptidoglycan-binding domain 1 protein	1.70E-48	Y	1
CC1G_14321	35	Hemolysin	9.81E-77	N	3
CC1G_08484	34	Cercosporin toxin biosynthesis protein	4.71E-115	N	N
CC1G_06959	31	Thaumatin-like protein	3.50E-79	Y	N
CC1G_13099	15	Peptidoglycan-binding domain 1 protein	4.40E-17	N	N
CC1G_05003	12	CGL1	7.13E-37	N	N
CC1G_11123	11	Toxin-antitoxin system, toxin component	1.84E-33	N	N
CC1G_11246	11	Ricin B-fold protein	7.70E-28	Y	N
CC1G_11805	11	Pore-forming protein	1.69E-40	N	N

*Fisher’s exact test.

†N or Y indicates the lack or presence of a signal peptide.

‡N indicates a lack of transmembrane helices, while a number corresponds to the amount of transmembrane helices predicted in the ORF.

**Table 3:** **VM-specific defense loci**

Locus	Fold VM/S1P	PSI Blast	*p-value	†SignalP	‡TMHMM
CC1G_05299	2076	Ricin B-fold protein	2.95E-240	N	N
CC1G_10614	1481	CFEM domain-containing protein	9.18E-166	Y	N
CC1G_15645	851	CFEM domain-containing protein	1.51E-194	Y	N
CC1G_09154	639	Cerato-platanin protein	6.24E-233	Y	N
CC1G_13813	527	CFEM domain-containing protein	6.90E-195	Y	N
CC1G_05638	67	Peptidoglycan-binding domain 1 protein	1.19E-119	Y	1
CC1G_05246	42	Ricin B-fold protein	7.71E-125	Y	N
CC1G_09155	24	Cerato-platanin protein	3.27E-75	Y	N
CC1G_09421	21	Terpenoid synthase	5.98E-27	N	N
CC1G_11847	17	Lysozyme	2.00E-08	Y	N
CC1G_08066	16	Ricin B-fold protein	1.01E-45	Y	N
CC1G_08310	13	Lysozyme	6.90E-09	Y	N
CC1G_15739	10	Ricin B-fold protein	1.54E-59	Y	N
CC1G_03046	8	Lysozyme	3.68E-05	Y	N

*Fisher’s exact test.

†N or Y indicates the lack or presence of a signal peptide.

‡N indicates a lack of transmembrane helices, while a number corresponds to the amount of transmembrane helices predicted in the ORF.

### The S1P-specific aerolysin/ETX pore-forming domain-containing protein CC1G_11805 is toxic to nematode and insect larvae

**Figure 2:**
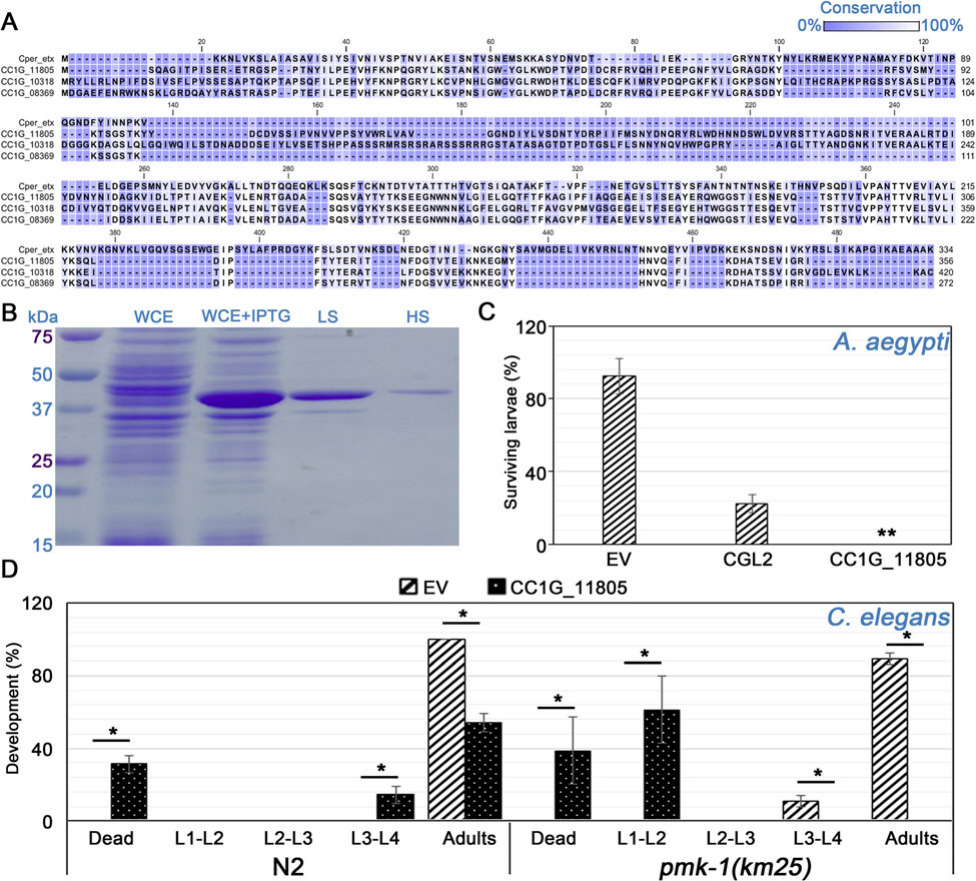
**The S1P-specific aerolysin/ETX pore-forming domain-containing protein CC1G_11805 is toxic for** ***C. elegans*** **and** ***A. aegypti*** **. (A)** Multiple sequence alignment of the aerolysin/ETX pore-forming domain-containing proteins *C. perfringens* epsilon toxin (Cper_etx), CC1G_11805, CC1G_10308 and CC1G_08369. **(B)** Expression of soluble CC1G_11805 in soluble form in *E. coli* BL21. Whole cell protein extracts in PBS were produced from non-induced (WCE) and induced (WCE + IPTG) *E. coli* BL21/pET24-CC1G_11805 cultures. Supernatants from consecutive low (LS) and high (HS) speed centrifugations were collected and run on a 12% SDS-PAGE to evaluate CC1G_11805 expression and solubility. CC1G_11805-mediated toxicity for *A. aegypti* **(C)** and *C. elegans* **(D)** larvae was assessed as described previously [40]. *E. coli* BL21 expressing the previously characterized fungal lectin CGL2 was used as positive control for toxicity against *A. aegypti* Rockefeller. IPTG-induced *E. coli* bearing an empty pET24 vector (EV) was used as a negative control. Columns represent the mean percentage of surviving insect larvae **(C)**, or worms either dying or reaching the indicated developmental stage **(D)** from 4 replicates each. SDs are shown as error bars. Dunn’s multiple comparisons were used to test the statistical significance of the toxicity observed in the *A. aegypti* assay. Mann Whitney test was used to compare the percentage of worms reaching each developmental stage when treated with EV or CC1G_11805. *: 0.01 < p-value < 0.05; **: 0.001 < p-value < 0.01.

CC1G_11805, CC1G_10318 and CC1G_08369 encode three homologous 30–40 kDa proteins containing a predicted aerolysin/ETX pore-forming domain (Figure 2A). This domain is homologous to the one present in the insecticidal epsilon toxin (ETX) from *Clostridium perfringens* [38] and distantly related to aerolysin pore-forming toxins [39]. In order to test the significance of the up-regulation of CC1G_11805 and CC1G_10318 in S1P with regard to fungal defense, we cloned and recombinantly expressed CC1G_11805 in *Escherichia coli* and assessed the toxicity of the protein by feeding the recombinant bacteria to nematode and insect larvae as described previously [40]. Results in Figure 2B show that CC1G_11805 was expressed in soluble form in *E. coli*. Feeding of CC1G_11805-expressing bacteria to L2 larvae of the mosquito *Aedes aegypti* lead to their death after 96 h (Figure 2C). Vector control-containing and fungal lectin-expressing *E. coli* were used as negative and positive controls, respectively, in these experiment [18, 29]. Similarly, feeding of CC1G_11805-expressing bacteria was found to significantly impair larval development of the *C. elegans* N2 wildtype and *pmk-1(km25)* mutant strains (Figure 2D); in agreement with the previously reported higher susceptibility of latter strain to different kinds of abiotic and biotic stresses including other nematotoxic fungal defense proteins [35], CC1G_11805 was shown to be more toxic to *C. elegans pmk-1(km25)* than to N2. These results support previous observations [18] showing that the expression of defense proteins directed against nematodes and insects is significantly increased in *C. cinerea* sexual organs.

### Differential expression of loci involved in sexual development of filamentous fungi

Various genes were recently described as playing a role at different stages of fruiting body formation of *C. cinerea* [10–17]. With the exception of the cyclopropane fatty acid synthase *cfs1*, none of these loci were found to be differentially transcribed during *C. cinerea* sexual development (Table 4, Additional file 6: Figure S2). The Velvet protein regulon plays a major role in the control of sexual vs. asexual development in the ascomycete *Aspergillus nidulans* [41, 42]; therefore, we took a closer look at the expression pattern of *C. cinerea* orthologs of the Velvet protein family and the proteins regulated by it [41, 42]. CC1G_12219, a Velvet domain-containing protein bearing an NLS is highly induced in S1P (S1P/VM = 237). In total, three out of six Velvet domain-containing proteins encoded in the *C. cinerea* genome (CC1G_12219, CC1G_06962 and CC1G_14883) were found to be developmentally regulated (Additional file 7: Figure S3). Among the other members of the Velvet protein regulon, two homologs of the VeA-regulated activator NsdD (CC1G_06391 and CC1G_06265) were also up-regulated in S1P (S1P/VM = 14 and 2.4, respectively). Moreover, the *C. cinerea* homeodomain transcription factor STE-12 (CC1G_02207) showed a moderate up-regulation in primordia (S1P/VM = 2). Finally, CC1G_07060, an ortholog of the *A. nidulans* repressor of sexual development *rosA*, was 34 fold up-regulated in VM compared to S1P. Taken together, the differential expression of several members of the Velvet protein regulon suggests that these genes may play a role in sexual development of basidiomycetes.

**Table 4:** **Most genes necessary for fruiting body formation in** ***C. cinerea*** **are not up-regulated in S1P**

Locus	Fold S1P/VM	Gene	*p-value	Mutant phenotype	Reference
CC1G_03287	0.8	*rmt1*	1.43E-01	No clamp connections	Nakazawa [15]
CC1G_00975	1.4	*ubc2*	1.75E-02	No hyphal knot formation	Nakazawa 2011
CC1G_15539	1.1	*snf5*	3.98E-01	Blocked at hyphal knot	Ando [17]
CC1G_11387	64.0	*cfs1*	5.25E-142	Blocked at initials	Liu [10]
CC1G_06825	1.2	*dst2*	9.66E-02	Blocked at stage 2 primordia	Kamada [16]
CC1G_08609	2.6	*dst1*	1.21E-13	Blocked at stage 2 primordia	Kamada [16]
CC1G_10193	1.5	*eln3*	1.40E-03	Blocked at immature mushroom	Arima [11]
CC1G_04713	2.3	*eln3*	5.18E-08	Blocked at immature mushroom	Arima [11]
CC1G_06451	2.5	*eln3*	9.03E-13	Blocked at immature mushroom	Arima [11]
CC1G_01334	0.8	*exp1*	3.65E-02	Blocked before mushroom decay	Muraguchi [12]

*Fisher’s exact test.

### Conserved transcriptional circuitry during fruiting body development among basidiomycetous fungi

**Figure 3:**
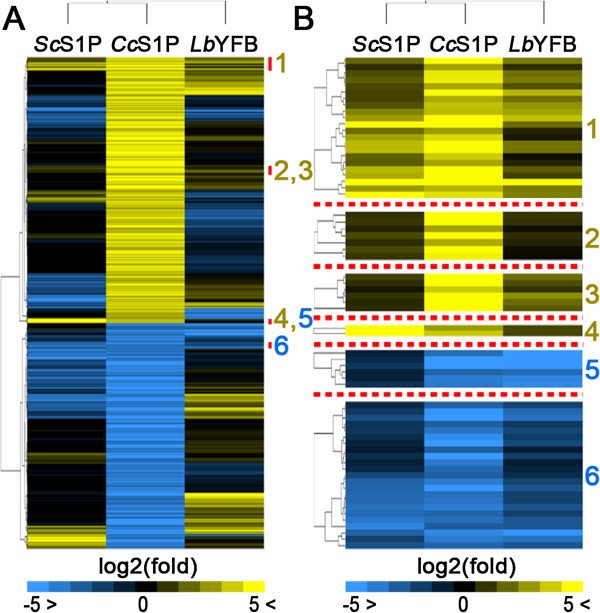
**Conserved expression pattern of some gene clusters during fruiting body formation among three different basidiomycetous species.** Comparative transcriptome analysis of early sexual development in *S. commune* 4-39/4-40, *C. cinerea* AB and *L. bicolor* S238N-H82 was performed. Orthologous genes (best PSI-BLAST hits with E-values ≤ 0.005) to only 446 and 378 up- and down-regulated loci in S1P, respectively, were found in *S. commune* 4-39/4-40 and *L. bicolor* S238N-H82. **(A)** Fruiting body transcriptome data were retrieved in the form of log2 ratios for these loci from the raw data sets and analyzed by centroid-linkage hierarchical clustering. The red sidelines mark the presence of 6 loci clusters which are consistently up (1–4)- or down (5 and 6)-regulated in young fruiting bodies (YFB) and stage 1 primordia (S1P) of the three species compared. **(B)** Close-up view of the up- and down-regulated clusters highlighted by red bars in **(A)**. Orthologous genes in *C. cinerea*, *S. commune* and *L. bicolor* corresponding to the numbered clusters, as well as the functional annotation of these loci and the cluster-associated Pearson correlation coefficients can be found in Table 5.

In order to explore a hypothetical conserved gene expression circuitry during the formation of SR structures in basidiomycetes, a comparative transcriptomic analysis by hierarchical clustering including *C. cinerea*, *S. commune* [43] and *L. bicolor* [44] was performed*.* Orthologous genes in *S. commune* and *L. bicolor* were assigned from a PSI-BLAST search (hits with the lowest E-value ≤ 0.005) to 446 and 378 up- and down-regulated loci in *C. cinerea* S1P, respectively. Massively parallel signature sequencing tags/million from *S. commune* monokaryotic mycelium and stage 1 primordia (*Sc*S1P); as well as *L. bicolor* free-living monokaryotic mycelium (2 replicates) and young fruiting bodies (*Lb*YFB, one replicate) robust multichip average values were retrieved from original studies [43, 44]. log2 expression ratios were calculated using monokaryotic mycelium expression values from these conserved loci as denominators and a centroid linkage hierarchical clustering analysis was computed (Figure 3; Additional file 8: Table S5). Four clusters corresponding to 37 up-regulated loci in early sexual development in basidiomycetes were identified (Pearson correlation coefficients: 0.95, 0.99, 0.99 and 0.99, respectively). In addition, two different gene groups (29 loci) were found to be down-regulated in early stages of fruiting body formation when compared to vegetative mycelium of the three species (Pearson correlation coefficients: 0.99 and 0.94, respectively) (Table 5).

**Table 5:** **Up- and down-regulation of orthologous loci is conserved in stage 1 primordia and young fruiting bodies of three basidiomycete species**

Cluster	***C. cinerea***locus	***S. commune***locus	***L. bicolor***locus	Functional annotation*
1(PC†: 0.95)	CC1G_01874	SCHCODRAFT_73392	LACBIDRAFT_301146	Hypothetical protein
	CC1G_02466	SCHCODRAFT_109665	LACBIDRAFT_297494	Hypothetical protein
	CC1G_00973	SCHCODRAFT_83399	LACBIDRAFT_293318	Chitin deacetylase
	CC1G_10475	SCHCODRAFT_57566	LACBIDRAFT_295571	Aromatic peroxygenase precursor
	CC1G_14095	SCHCODRAFT_48101	LACBIDRAFT_294461	Hypothetical protein
	CC1G_03094	SCHCODRAFT_52606	LACBIDRAFT_315775	Hypothetical protein
	CC1G_06074	SCHCODRAFT_76530	LACBIDRAFT_293318	Carbohydrate esterase family 4 protein
	CC1G_10044	SCHCODRAFT_108884	LACBIDRAFT_306386	Related to S.pombe pac2 protein
	CC1G_00753	SCHCODRAFT_234371	LACBIDRAFT_305130	Hypothetical protein
	CC1G_14786	SCHCODRAFT_52306	LACBIDRAFT_246776	MFS nicotinic acid transporter Tna1
	CC1G_01301	SCHCODRAFT_72462	LACBIDRAFT_309640	Hypothetical protein
	CC1G_05059	SCHCODRAFT_52448	LACBIDRAFT_308735	Symbiosis-related protein
	CC1G_11437	SCHCODRAFT_67374	LACBIDRAFT_317173	Putative aquaporin 6
	CC1G_08178	SCHCODRAFT_237423	LACBIDRAFT_323328	Thioesterase family protein
	CC1G_08180	SCHCODRAFT_237423	LACBIDRAFT_323328	Thioesterase
	CC1G_01879	SCHCODRAFT_257636	LACBIDRAFT_326397	mei2 protein Piriformospora
	CC1G_05223	SCHCODRAFT_70203	LACBIDRAFT_250946	DUF1275 domain protein
	CC1G_12219	SCHCODRAFT_28806	LACBIDRAFT_317102	Related to velvet A protein
	CC1G_10750	SCHCODRAFT_86141	LACBIDRAFT_184665	Glutathione S-transferase
	CC1G_04060	SCHCODRAFT_13677	LACBIDRAFT_180892	Hydrophobin-251
	CC1G_10471	SCHCODRAFT_232646	LACBIDRAFT_295571	Aromatic peroxygenase precursor
	CC1G_.12330	SCHCODRAFT_16019	LACBIDRAFT_256021	S-layer domain-containing protein
2 (PC: 0.99)	CC1G_00069	SCHCODRAFT_48115	LACBIDRAFT_312065	C factor cell-cell signaling protein
	CC1G_00217	SCHCODRAFT_46102	LACBIDRAFT_323571	Non-Catalytic module family EXPN protein
	CC1G_03515	SCHCODRAFT_82058	LACBIDRAFT_328112	Hydrophobin-like protein
	CC1G_04659	SCHCODRAFT_110821	LACBIDRAFT_298314	Hypothetical protein
	CC1G_05652	SCHCODRAFT_232455	LACBIDRAFT_309898	TKL/TKL-ccin protein kinase
	CC1G_09209	SCHCODRAFT_110821	LACBIDRAFT_298284	BTB/POZ domain containing protein
	CC1G_10966	SCHCODRAFT_258323	LACBIDRAFT_236299	rCop c3
3 (PC: 0.99)	CC1G_02571	SCHCODRAFT_76530	LACBIDRAFT_293318	Chitin deacetylase
	CC1G_03037	SCHCODRAFT_53388	LACBIDRAFT_239714	Monocarboxylate transporter
	CC1G_03320	SCHCODRAFT_39718	LACBIDRAFT_145110	Spo11
	CC1G_09616	SCHCODRAFT_235946	LACBIDRAFT_310809	Prenyl cysteine carboxyl methyltransferase
	CC1G_06563	SCHCODRAFT_46720	LACBIDRAFT_243581	Exo-beta-1,3-glucanase
	CC1G_12688	SCHCODRAFT_55636	LACBIDRAFT_296585	Aldo/keto reductase
4 (PC: 0.99)	CC1G_03949	SCHCODRAFT_258034	LACBIDRAFT_298461	Salicylate hydroxylase
	CC1G_05817	SCHCODRAFT_258034	LACBIDRAFT_298461	Salicylate 1-monooxygenase
5 (PC: 0.99)	CC1G_06868	SCHCODRAFT_53362	LACBIDRAFT_182606	Endo-1,3(4)-beta-glucanase
	CC1G_07550	SCHCODRAFT_56140	LACBIDRAFT_291657	Lipase
	CC1G_05781	SCHCODRAFT_72461	LACBIDRAFT_291413	OrdA protein
	CC1G_08860	SCHCODRAFT_80181	LACBIDRAFT_172283	Cation/H + exchanger
	CC1G_09431	SCHCODRAFT_49922	LACBIDRAFT_190903	O-methylsterigmatocystin oxidoreductase
	CC1G_11000	SCHCODRAFT_107054	LACBIDRAFT_310382	Hypothetical protein
6 (PC: 0.94)	CC1G_02440	SCHCODRAFT_103563	LACBIDRAFT_308947	ras GEF
	CC1G_07544	SCHCODRAFT_80832	LACBIDRAFT_243185	DUF89-containing protein
	CC1G_05410	SCHCODRAFT_104205	LACBIDRAFT_301153	Hypothetical protein
	CC1G_02628	SCHCODRAFT_82883	LACBIDRAFT_327335	C2h2 conidiation transcription factor FlbC
	CC1G_11894	SCHCODRAFT_68168	LACBIDRAFT_296675	bas1, putative
	CC1G_05329	SCHCODRAFT_85265	LACBIDRAFT_300118	Hypothetical protein
	CC1G_09061	SCHCODRAFT_27314	LACBIDRAFT_308057	MNNG and nitrosoguanidine resistance protein
	CC1G_02380	SCHCODRAFT_77933	LACBIDRAFT_314347	Clavaminate synthase-like protein
	CC1G_05793	SCHCODRAFT_85265	LACBIDRAFT_300118	Hypothetical protein
	CC1G_14014	SCHCODRAFT_49922	LACBIDRAFT_248655	O-methylsterigmatocystin oxidoreductase
	CC1G_11136	SCHCODRAFT_232299	LACBIDRAFT_294457	Hypothetical protein
	CC1G_00780	SCHCODRAFT_106836	LACBIDRAFT_305061	BGP, partial
	CC1G_03120	SCHCODRAFT_83385	LACBIDRAFT_307143	Endoglucanase II
	CC1G_02257	SCHCODRAFT_46132	LACBIDRAFT_295167	Metalloprotease
	CC1G_04116	SCHCODRAFT_75425	LACBIDRAFT_179508	Glutathione S-transferase Gst3
	CC1G_11081	SCHCODRAFT_75425	LACBIDRAFT_179508	Glutathione S-transferase
	CC1G_12752	SCHCODRAFT_50449	LACBIDRAFT_253329	Cytochrome P450
	CC1G_12253	SCHCODRAFT_269973	LACBIDRAFT_296037	Nrg1-like Zn-finger transcription factor
	CC1G_11786	SCHCODRAFT_58473	LACBIDRAFT_315853	Hypothetical protein
	CC1G_09938	SCHCODRAFT_80558	LACBIDRAFT_330070	Ferritin/ribonucleotide reductase-like-protein
	CC1G_02724	SCHCODRAFT_255861	LACBIDRAFT_295183	Hypothetical protein
	CC1G_05802	SCHCODRAFT_65657	LACBIDRAFT_256254	Indole-3-acetaldehyde dehydrogenase
	CC1G_15739	SCHCODRAFT_76887	LACBIDRAFT_146952	Ricin B fold protein

*Functional annotation as determined by PSI-BLAST.

†Pearson correlation coefficient.

This hierarchical clustering approach of gene expression in orthologous loci during sexual development showed that loci involved in core meiotic functions, such as *mei2* (CC1G_01879) and *spo11* (CC1G_03320), are up-regulated during the early stages of fruiting body formation in basidiomycetes.

Given the up-regulation of a conserved Velvet-domain containing protein in primordia of the analyzed basidiomycetes, the conservation and expression pattern of Velvet-interacting proteins previously characterized in *Aspergillus* [42] was examined. With the exception of *cryA*, orthologs to these Velvet-interacting proteins can be found in *C. cinerea*, *L. bicolor* and *S. commune* (Additional file 9). Contrary to the conserved transcriptional regulation observed for genes encoding Velvet domain-containing proteins, little conservation in expression is evident for the orthologs to *rosA*/*nosA*, *stuA*, *nsdD*, *ppoA*, *laeA* or *fphA* (Additional file 10: Figure S4). Nevertheless, conserved down-regulation during sexual development is shown for *velB* and *kapA*, suggesting that a fraction of the Velvet-associated regulon described for ascomycetes might be playing a role during sexual development in basidiomycetes (Additional file 10: Figure S4). Similarly, down-regulation of transcription factors such as *flbC* (a paralog to C2H2 known to regulate development in *A. nidulans* [45]), *nrg1* and *bas1* during early mushroom formation was conserved between the three basidiomycetous species.

Transcription factor mutants (Bri1, Hom1, Gat1, Fst3, C2h2, Fst4 and Hom2) altering normal sexual development in *S. commune* have been described [46, 47]. Although orthologs of these transcription factors are present in *C. cinerea* and *L. bicolor*, the respective genes do not show a conserved expression pattern, indicating that there might be differences in the interplay between these factors during fruiting body formation between different basidiomycetes (Additional file 11: Figure S5).

### Shotgun MS analysis of PBS-soluble VM and S1P proteins

In order to confirm protein expression of some of the genes found to be differentially expressed by RNA-seq, we assessed the PBS-soluble *C. cinerea* proteome in VM and S1P by LC-MS. The analysis detected peptides corresponding to a total of 493 proteins in the samples, including 41, 141 and 311 proteins in S1P, VM or both, respectively (Additional file 12: Table S6). This analysis is highly biased towards abundant and soluble proteins and is likely to have failed to detect peptides from most of the loci identified by RNA-seq. Nevertheless, this method allowed us to confirm the translation of 12 transcripts up-regulated in S1P and 50 transcripts up-regulated in VM into proteins in the respective tissues (Additional file 13: Table S7).

## Discussion

Our data shows a transcriptional switch during the differentiation of primordia in *C. cinerea* comprising 11% of the protein-encoding genome being up- or down-regulated. Differential transcription analyses carried out in other basidiomycetous fungi, such as *Agrocybe aegerita*, *Cordyceps militaris* and *Ganoderma lucidum*, have revealed similar or even more extensive transcriptional switches [48–50]. Taking the differential expression threshold used in the present study (log2(fruiting body/vegetative mycelium) ≥ 3), 25% and 30% out of 18474 *A. aegerita* loci are up-regulated in fruiting bodies and vegetative mycelium, respectively [48]. Similarly, *C. militaris* up-regulates 40% loci during fruiting body formation [49], while *G. lucidum* boosts the transcription of at least 27% measured loci in its sexual organs [50].

The enrichment among the up-regulated loci in S1P of the functional annotation tag “N-methyltransferase” (histone-lysine N-methyltransferase, 7 loci in total) suggests that epigenetic regulation of gene expression or chromosome dynamics may play an important role during sexual development in *C. cinerea*. In *Saccharomyces cerevisiae*, histone H3 lysine 4 trimethylation marks the sites where double-strand breaks, the first events during interhomolog recombination, occur [51]. Up-regulation of histone-lysine N-methyltransferases in S1P is in accordance with karyogamy and early meiotic phases taking place at stage 1 primordia during fruiting body formation [6].

Several previously characterized nematotoxic and insecticidal proteins [18, 20] were found to be up-regulated in S1P. Among these proteins, lectins appear to be most abundant. The fruiting body-specific ricin B-like lectins CCL1 and CCL2 were previously shown to be toxic to *C. elegans* due to the binding to α1,3-fucosylated N-glycan cores in the intestine of L1 worms [34]. In accordance to a previous study [52], we found that the tetrameric galectin CGL1 (CC1G_05003) was highly induced in *C. cinerea* fruiting bodies. Similar to CCL1 and CCL2, CGL1 and its isogalectin CGL2 (CC1G_05005) showed toxicity against *C. elegans* which was dependent on binding to a Galβ1,4Fucα1,6-epitope on N-glycan cores present in the worm intestine [35]. Protease inhibition is another defense strategy against predation in *C. cinerea* fruiting bodies. We found the locus encoding the serine protease inhibitor Cospin1 (CC1G_09480) and its isoprotein Cospin2 (CC1G_09479) among the top 50 most highly transcribed and differentially expressed loci in S1P. Cospin1 was previously shown to be toxic against *Drosophila melanogaster* larvae indicative of its role in fruiting body defense against arthropod predation [20]. In this study, we demonstrate that CC1G_11805 is toxic for *C. elegans* and *A. aegypti* larvae, analogous to the toxicity of aerolysin/ETX pore-forming domain-containing *Bacillus sphaericus* protein towards larvae of the mosquito *Culex quinquefasciatus* [53], suggesting that pore formation is another defense strategy of *C. cinerea* primordia against predators. The high absolute transcription of these defense loci in S1P can be explained by the extensive resource allocation to these organs and the significance of these organs for reproduction of the fungus. The expression of putative secreted and antibacterial proteins in *C. cinerea* vegetative mycelium, on the other hand, probably reflects the confrontation of this tissue with bacterial competitors although the antibacterial activity of these proteins has still to be demonstrated. In summary, the differential expression of defense proteins in the different tissues of *C. cinerea* is an adaptation of the fungus to the different environmental challenges with which these tissues are confronted.

Surprisingly, with the exception of *cfs1*, none of the genes previously shown to play a role in *C. cinerea* fruiting body formation were differentially expressed in stage 1 primordia. Mutations in *rmt1*, *ubc2* and *snf5* [14, 15, 17] block development before initials are formed (48 h before S1P develop). In contrast, *dst1*, *dst2*, *eln3* and *exp1* are involved in processes taking place in stage 2 primordia (24 h after S1P), immature fruiting bodies (48–72 h after S1P) and decaying fruiting bodies (72 h after S1P) [6, 11, 12, 16]. Thus, a possible explanation of our results is that these genes might be regulated before or after the formation of S1P takes place. Intriguingly, the mutation of *cfs1* blocks development right at the transition between initials and S1P. This observed up-regulation of *cfs1* in S1P supports a function for this cyclopropane fatty acid synthase in the development of S1P from initials.

The Velvet protein regulon, including genes *nsdD*, *rosA*, *veA* and *stuA* among others, coordinates sexual development and secondary metabolism in filamentous ascomycetes [41, 42]. Velvet domains structurally resemble the RHD-like fold present in the transcription factor NF-κβ that plays a central role in animal immunity, suggesting a common evolutionary origin for these two protein families [54]. Overexpression of *veA* and *nsdD* in *A. nidulans* induces the formation of nursing Hülle cells surrounding the cleistothecia [41]. Homologous genes in *C. cinerea*, CC1G_12219 and CC1G_06391, showed high expression in S1P suggesting a function of these loci in the gene circuitry involved in fruiting body development in this fungus. On the other hand, *rosA*, an *A. nidulans* transcription factor inhibiting sexual development in low-carbon culture, is expressed in *A. nidulans* asexual hyphae where it represses the transcription of sexual development regulators such as *nsdD*, *veA* and *stuA* [55]. Similarly, *rosA*-homologous *C. cinerea* gene CC1G_07059, is expressed in vegetative mycelium where it might be inhibiting sexual development. Taken together, these results suggest a conserved role of the Velvet protein regulon during sexual development in the ascomycete *A. nidulans* and the basidiomycete *C. cinerea*.

Genes encoding Velvet domain-containing proteins were also found to be up-regulated in fruiting bodies of *S. commune* and *L. bicolor* [43, 44]. However, (with the exception of *velB* and *kapA*) genes encoding Velvet-associated proteins such as RosA/NosA, StuA, NsdD, PpoA, LaeA, FphA or CryA [42], do not show inter-species conservation of differential expression. Similar lack of transcriptional conservation between the three analyzed basidiomycete species was observed for orthologs to transcription factors shown to be involved in *S. commune* sexual development [46, 47]. Taken together, this evidence suggests that basidiomycetes show species-specific divergence of transcriptional regulation in orthologous genes similar to plants [56].

A broader comparison of the transcriptomes of the three analyzed basidiomycete species, comprising all the genes differentially expressed in *C. cinerea* during sexual development, revealed the presence of a conserved gene regulation circuitry among basidiomycetes during fruiting body formation. As previously observed in fruiting bodies of ascomycetes [57], our orthology analysis showed that a large fraction of genes differentially expressed in S1P corresponds to loci which are not present in the basidiomycetes *L. bicolor* and *S. commune*. These results are in agreement with previous observations in plants and animals that genes associated with sexual reproduction rapidly evolve [58, 59]. The existence of clusters comprising conserved up- or down-regulated loci with little inter-species expression variability, suggests that regulation of these loci evolved under stabilizing selection [60]. Sequence and expression conservation might imply an essential role of these genes in fruiting body development and sexual reproduction in basidiomycetes. Interestingly, *L. bicolor* and *C. cinerea* orthologs to transcription factors described previously as important for sexual development in *S. commune*, such as Bri1, Hom1, Gat1, Fst3, C2h2, Fst4 or Hom2 [46, 47], do not show conserved transcriptional conservation in basidiomycetes, suggesting that novel regulatory pathways related to sexual development did evolve once speciation occurred. As a proof of concept, Traeger and collaborators found that the fruiting body specific transcription factor *pro44* (orthologous to the velvet regulon-associated transcription factor *nsdD*) in the ascomycetes *Sordaria macrospora* and *Pyronema confluens* is a core regulator of perithecia maturation. *S. macrospora* deficient in *pro44* was shown to be sterile and unable to produce mature perithecia [57]. In addition to a Velvet domain-containing protein, the expression of the RNA binding protein *mei2* (CC1G_01879), a master meiosis regulator in yeast and plants [61, 62] protecting meiosis-specific transcripts from degradation by the DSR-Mmi system, was increased in S1P or YFB of *C. cinerea*, *S. commune* and *L. bicolor*. Similarly, the transcriptional regulation of *spo11*, encoding a protein inducing meiotic recombination in *S. cerevisiae* and *C. cinerea* [63], was also conserved. This induction of meiosis regulators in multiple species reflects the role of mushrooms in the production and dispersal of basidiospores.

With regard to the comparative transcriptome analysis of the three basidiomycete species, it should be noted that in case of *S. commune* and *L. bicolor*, only data of monokaryotic vegetative mycelia was available [43, 44], whereas in case of *C. cinerea*, the transcriptome of an isogenic homodikaryotic mycelium was determined. Thus, the degree of conserved transcriptional regulation during sexual development between *C. cinerea*, *L. bicolor* and *S. commune* is potentially larger than observed.

Lastly, the proteome of an organism provides a more direct image of its phenotype than the transcriptome [64]. Detection of proteins using label-free shotgun mass spectrometry fails to detect low abundance proteins in complex total extracts and allows only semi-quantitative estimation of relative protein amounts. These properties are in contrast to the superior standardization and sensitivity achieved by state of the art nucleotide sequencing technologies. Nevertheless, LC-MS spectra showed the presence of peptides derived from the nematotoxic lectin CCL1 (CC1G_11778) and the nematotoxic/insecticidal aerolysin/ETX pore-forming domain-containing protein CC1G_11805 in the PBS-soluble protein extract of *C. cinerea* stage 1 primordia, indicating that these cytoplasmic toxins are indeed expressed at protein level in these organs.

The recent development of gene targeting tools in *C. cinerea* [13, 65], will help to test whether some of the genes identified as differentially regulated during sexual development in multiple basidiomycete species, play a role in this process.

## Conclusions

In this work, we show that sexual reproduction in *C. cinerea* A43mutB43mut involves the differential transcription of at least 11% of its protein-coding genome. Differentially transcribed genes include several genes coding for defense proteins that protect fruiting structures and vegetative mycelia from predators and competitors, respectively. Moreover, our data infers a role of the Velvet protein family during fruiting body formation in basidiomycetes and thus, a conserved role of this protein family during sexual development in dikaryotic filamentous fungi. Finally, the result of the comparative transcriptome analysis of *C. cinerea*, *S. commune* and *L. bicolor* suggest that a conserved set of orthologous genes regulates sexual development in the phylum Basidiomycota. Additional experiments addressing the function of the differentially expressed gene products are required to confirm these hypotheses.

## Methods

### Strains and culture conditions

The dikaryotic, self-compatible *C. cinerea* strain A43mutB43mut (AB) [66] was grown on 30 mL YMG plates (0.4% yeast extract, 1% malt extract, 50 mM glucose and 1.5% agar) at 37°C in the dark for 96 h and transferred to 25°C, 90% humidity and 12 h photoperiod for fruiting body production. 1 to 2 mm AB S1P [6] were harvested after 72 h, flash-frozen in liquid nitrogen and stored at -80°C for later use. *C. cinerea* AB VM was grown in duplicate on YMG plates covered with cellophane discs for 96 h at 37°C in the dark and harvested independently before being flash frozen and stored at -80°C.

### Total RNA extraction

Primordia and mycelia were lyophilized and S1Ps were separated in two pools of 20 mg each. From each VM replicate or S1P pool, 20 mg dry material were lysed in three FastPrep FP120 homogenization steps of 45 s at 4.5, 5.5 and 6.5 m/s in the presence of 250 mg 0.5 mm glass beads, cooling the samples for 5 min on ice between steps. RNA was extracted using 1 mL Qiazol (Qiagen) and 0.2 mL chloroform ReagentPlus (Sigma-Aldrich). The solution was centrifuged at 12000 × g for 15 min at 4°C; thereafter, RNA from the resultant aqueous phase was washed on-column using the RNeasy Lipid Tissue Mini Kit (Qiagen) and eluted in 60 μL RNase-free water. Concentration and integrity of the purified RNA were determined with a Qubit (1.0) fluorometer (Life Technologies) and a Bioanalyzer 2100 (Agilent), respectively. Samples with a 260/280 nm ratio of 1.8–2.1 and a 28S/18S ratio of 1.5–2 were later used in library construction.

### SOLiD 4 library construction

Whole transcriptome libraries from two S1P pools and two VM replicates were produced using MicroPolyA Purist Kit (Ambion) and SOLiD Total RNA-Seq kit (Applied Biosystems). Briefly, approximately 200 ng/sample poly (A)-positive RNA was enriched using MicroPoly (A) Purist Kit from 15–20 μg total RNA. Quality and concentration of the extracted poly (A)-positive RNA was re-assessed as described above, and poly (A)-positive RNA was digested with RNase III. Ligation of the adaptor mix and reverse transcription was performed following the manufacturer instructions. cDNA libraries were size selected for 150–250 bp fragments, amplified in 15–18 PCR cycles using barcoded adaptor primers and purified with PureLink PCR micro kit (Invitrogen). These barcoded cDNA libraries were then amplified by emulsion PCR from 0.5 pM template. Sequencing beads from the barcoded libraries were pooled and loaded on a SOLiD 4 slide (Applied Biosystems), according to manufacturer’s instructions. SOLiD ToP Sequencing chemistry was used to produce pair end (50 bp + 35 bp) sequencing reads.

### qRT-PCR validation

RNA-seq results were validated by qRT-PCR. Single-stranded cDNA from one biological replicate per sample was synthesized using Transcriptor Universal cDNA Master (Roche) from 2 μg total RNA. 20 μL qRT-PCR reactions were mixed in three technical replicates per primer set and sample, containing 900 nM forward and reverse primers designed to span exon-exon junctions (Additional file 14: Table S8), 10 μL 2× FastStart Universal SYBR Green Master (Rox, Roche) and 1 ng/μL cDNA template. qRT-PCR was performed in a Rotor-Gene 3000 (Corbett Life Science) with the following thermal profile: a hold step at 95°C for 15 min followed by 40 cycles of 95°C for 15 s, 58°C for 30 s and 72°C for 30 s. In order to control the specificity of amplification, the reaction was concluded with a melting curve analysis ramping from 55°C to 99°C in steps of 1°C every 5 s. PCR efficiencies and cycle thresholds were obtained using LinRegPCR 12 [67] and differential expression ratios were calculated by the C_T_ difference formula [68]. Tubulin beta chain (CC1G_04743) was used as a house keeping normalizer. In addition, water or 1 ng/μL RNA were included as negative control reactions. To further validate the significance of the RNA-seq-derived differential expression analysis, the constitutive expression of an array of housekeeping loci commonly used in qRT-PCR normalization [69–75] was verified in the sequencing datasets after library size normalization.

### Bioinformatic analysis

Fastqc files were used to filter and trim the reads to be mapped. Strand-specific reads were mapped to the third annotation (September 2010) of the *C. cinerea* okayama7#130 genome hosted at the Broad Institute (*C. cinerea* Sequencing Project, Broad Institute of Harvard and MIT (http://www.broadinstitute.org/)). SOLiD mapped reads were counted using Cufflinks [76]. All the sequences were deposited in the ArrayExpress database (http://www.ebi.ac.uk/arrayexpress/) under the accession number [E-MTAB-1968]. To determine the percentage of loci showing baseline expression, 5 reads/locus were taken as a minimal threshold [77]. Library size normalization (scaling method), fold change calculation per locus and fisher exact test comparing mycelia and primordia libraries were performed using the edgeR package [78] using sense-read counts. Fisher exact test p-value ≤ 0.05 and fold change ≥ 8 were the criteria established to classify a locus as differentially expressed in one of the two compared samples. Functional annotation of loci found to be developmentally regulated was explored by PSI-BLAST [79]. Enrichment of annotation terms in differentially expressed loci was visualized in tag clouds constructed by Wordle (© IBM Corporation) [32]; common tags such as “protein”, “hypothetical”, “domain”, “containing”, “family”, “putative”, “similar”, “probable”, “related” and “subunit” were excluded. A complementary functional annotation clustering was performed with the Database for Annotation, Visualization and Integrated Discovery (DAVID) 6.7 [33] using default annotation categories and the *C. cinerea* okayama7#130 translated genome as background. SignalP 4.1 [80] and TMHMM v. 2.0 [81] were used to predict the presence of secretion signal and transmembrane helices in developmentally regulated loci. Presence of nuclear localization signals (NLSs) in members of the Velvet protein family was assessed with NLStradamus [82]. To analyze the similarity in the gene expression programs associated to early stages in fruiting body development across different basidiomycetous fungi, orthologous genes to differentially expressed loci in *C. cinerea* S1P were identified by PSI-BLAST (best hit showing an E-value ≤ 0.005) in the genomes of *Schizophyllum commune* 4-39/4-40 and *Laccaria bicolor* S238N-H82. Expression data were retrieved from the original studies where the gene expression of vegetative mycelium and S1P or YFB from *S. commune* [43] and *L. bicolor* [44], respectively, was measured. First, log2 expression ratios were calculated for stage 1 primordia against monokaryon mycelium from *S. commune* using tags per million per locus. Similarly, log2 ratios were calculated for “young fruiting bodies” compared to “free living mycelium” (the latter corresponding to the average of two replicates) of *L. bicolor* from quantile-normalized Robust multichip average values. A centroid-linkage hierarchical clustering analysis of the computed log2 ratios was performed with Cluster 3.0 and heat maps were generated and visualized using TreeView 3 [83].

A more detailed orthology and expression analysis was carried out for loci known to encode proteins interacting with Velvet domain-containing proteins involved in sexual development and secondary metabolite production in *Aspergillus* [42]. The sequences of RosA, NosA, StuA, NsdD, PpoA, VelB, LaeA, KapA FphA and CryA from *A. nidulans* were used to retrieve the corresponding orthologs from the protein-coding genomes of *C. cinerea* Okayama 7, *S. commune* 4-39/4-40 and *L. bicolor* S238N-H82. Orthologs were defined as the best hits in a PSI-BLAST showing E-values ≤ 0.005. Progressive multiple sequence alignments for the orthologs present in *A. clavatus*, *A. fumigatus*, *A. oryzae* and *A. nidulans*, as well as in the three basidiomycetous species considered, were constructed in CLUSTAL W [84]. Finally, Velvet domain-containing proteins in *C. cinerea*, *S. commune* and *L. bicolor* were identified in Pfam and SMART diagrams were created [85].

### LC-MS/MS based shotgun proteomics

Twenty mg lyophilized VM from a single plate or a S1P pool were lysed in a FastPrep FP120 during 40 s at 6 m/s using 200 mg 0.5 mm glass beads and 600 μL PBS supplemented with 1 mM phenylmethanesulfonylfluoride (PMSF) and 1× Complete Protease Inhibitor Cocktail (Roche). The lysates were centrifuged for 15 min at 16000 xg and 4°C, and the supernatants (soluble protein extracts) recovered. Soluble protein extracts from VM and S1P were further processed by filter-aided sample preparation (FASP) method as previously described [86]. In brief, protein extracts were loaded onto an Amicon column equipped with a 10 kDa MWCO membrane (Millipore), reduced with 55 mM dithiothreitol at 37°C for 1 h and alkylated with 65 mM iodoacetamide in the dark at 37°C for 1 h. Reduced and alkylated extracts were digested with sequencing grade porcine trypsin (Roche) for 18 h at 37°C in 25 mM ammonium bicarbonate, pH 8.5. Digested peptide mixtures were collected by centrifugation and dried in a Savant SpeedVac (Thermo Scientific). All samples were de-salted by C18 ZipTip before mass spectrometry analysis.

Samples were analyzed on a LTQ-Orbitrap Velos mass spectrometer (Thermo Fischer Scientific) coupled to an Eksigent-Nano-HPLC system (Eksigent Technologies). Peptides were suspended in 2.5% acetonitrile and 0.1% formic acid, loaded on a self-made tip column (75 μm × 80 mm) packed with reverse phase C18 material (AQ, 3 μm 200 Å, Bischoff GmbH) and eluted with 250 nL/min flow rate in a gradient from 3% to 50% of B in 90 min, 97% B in 10 min. One scan cycle comprised a full scan MS survey spectrum, followed by up to 20 sequential CID MS/MS on the most intense signals above a threshold of 1500. Full-scan MS spectra (300–2000 m/z) were acquired in the FT-Orbitrap at a resolution of 60000 at 400 m/z, while CID MS/MS spectra were recorded in the linear ion trap. CID was performed with a target value of 1E4 in the linear trap, collision energy at 35 V, Q value at 0.25 and activation time at 30 ms. AGC target values were 5E5 for full FTMS scans and 1E4 for ion trap MSn scans. For all experiments, dynamic exclusion was used with one repeat count, 15 s repeat duration, and 60 s exclusion duration. For quantitation, each sample was measured in technical triplicates using the same parameters.

All MS data from VM and S1P were converted to a peak list and searched against the *C. cinerea* okayama7#130 proteome (predicted transcript translation) hosted at the Broad Institute (*C. cinerea* Sequencing Project, Broad Institute of Harvard and MIT (http://www.broadinstitute.org/)) using the Mascot search engine (version 2.3) considering variable modifications: carbamidomethylation on cysteine and oxidation on methionine. The tolerance of mass accuracy of MS and MS/MS was 8 ppm and 0.6 Da. The false discovery rate of proteome dataset was 1% and the score of each protein exceeded 30.

### Cloning and recombinant expression of CC1G_11805-encoding cDNA

cDNA encoding CC1G_11805 was amplified from *C. cinerea* S1P-derived cDNA using the forward and reverse primers 5′-CCAGCTTAAAGGAGTCACAAGG-3′ and 5′-AACGTTCAACGCCCAGCCAC-3′, respectively, with a Pfu DNA polymerase. 3′ adenines were added to the PCR fragment before ligation to the DNA amplification vector pGEM-T Easy (Promega). This pGEM-CC1G_11805 construct was used as a template to add NdeI and BamHI restriction sites using the primers 5′-GGGGGGCATATGTCTCAAGCAGGGATCACAC-3′ and 5′-GGGGGGGGATCCTCAGATACGCCCGATGACTTC-3′, respectively. The resulting product was digested with NdeI and BamHI in 2× Tango buffer (Thermo Scientific), ligated to a pre-digested pET24 expression vector (EMD Millipore), and used to transform chemocompetent *E. coli* BL21. Transformed colonies were selected on LB plates containing 50 μg/mL Kanamycin. CC1G_11805 recombinant expression and solubility were assessed as follows: pET24-CC1G_11805-containing *E.coli* BL21 was grown in LB broth supplemented with 50 μg/mL Kanamycin up to OD_600_: 0.5; thereafter, the cultures were divided in two separated flasks and protein expression was induced for 18 h at 24°C by adding 1 mM Isopropyl β-D-1-thiogalactopyranoside (IPTG, final concentration) to only one of the two subcultures. Cells were harvested, suspended in 1 mL PBS containing with 1 mM phenylmethanesulfonylfluoride (PMSF) and lysed in a single FastPrep FP120 homogenization step of 35 s at 6 m/s in the presence of 1 g 0.1 mm glass beads. The whole cell protein extract (WCE) was centrifuged for 5 min at 5000 xg and 4°C (low speed centrifugation, LS) to remove cell debris and the supernatant transferred to a fresh tube to be centrifuged again for 30 min at 14000 xg and 4°C (high speed centrifugation, HS). Samples were collected from the whole cell extracts (WCE and WCE + IPTG) and the two centrifugation supernatants (LS and HS), mixed with Laemmli buffer and denatured at 95°C for 5 min to be loaded on a 12% SDS-PAGE.

### *Caenorhabditis elegans*toxicity assay

In order to evaluate the developmental toxicity of CC1G_11805 to nematodes, a *C. elegans* toxicity assay was performed as previously described [40]. In brief, *C. elegans* N2 and *C. elegans pmk-1 (km25)* (kindly provided by M. O. Hengartner) were grown on NGM plates (50 mM NaCl, 2.5 g/L bacteriological peptone, 13 mM cholesterol and 1.7% agar) pre-seeded with *E. coli* OP50. Eggs were obtained by bleaching gravid hermaphrodites in 15 mL conical bottom tubes using a solution containing 0.5 N NaOH and 1% NaClO for 10 min and washing twice in 10 mL distilled deionized water (ddH_2_O). Clean eggs were transferred to a 1.5% agar plate and hatched for 18 h at 20°C. L1 larvae were collected in PBS, counted and adjusted to 1.5 larvae/μL. Approximately 30 L1 larvae/replicate (4 replicates/treatment) were mixed in 200 μL PBS with OD_600_: 2 IPTG-pre-induced *E. coli* BL21 expressing CC1G_11805 in flat bottom 96 well-plates. As a negative control, IPTG-pre-induced *E. coli* BL21 cells transformed with empty vector pET24 were used. Worms were incubated for 48 h at 20°C and the number of individuals reaching each developmental stage or dying was counted. A Mann Whitney test between the empty vector pET24 control and CC1G_11805 was run to test the statistical significance of the results observed.

### *Aedes aegypti*toxicity assay

CC1G_11805 toxicity towards *A. aegypti* Rockefeller (kindly provided by W. Rudin and P. Müller) was tested as previously described [40]. In brief, 600–800 eggs were hatched in a glass petri dish containing 200 mL deionized water and 30 mg ground fish food for 20 h at 28°C in the dark. Larvae were transferred to fresh 800 mL deionized water supplemented with 50 mg ground fish food and incubated for 10 h at 28°C in order to obtain synchronized L2 larvae. Ten L2 larvae/replicate (4 replicates/treatment) were starved in 100 mL fresh deionized water in 100 mL Schott flasks for 6 h at 28°C before adding 1 mL OD_600_: 20 (Final OD_600_: 0.2) IPTG-pre-induced *E. coli* BL21 (empty vector pET24, pET24-CGL2 [18] or pET24-CC1G_11805). Larvae were incubated for 96 h at 28°C in the dark and the surviving individuals were counted. Percentages of surviving larvae/treatment were calculated. A Dunn’s multiple comparison test between the empty vector pET24 control and the different treatments was run to test the statistical significance of the results observed.

### Availability of supporting data

The data sets supporting the results of this article are available in the ArrayExpress repository under the accession number E-MTAB-1968 (http://www.ebi.ac.uk/arrayexpress/).

## Electronic supplementary material

Additional file 1: Table S1: Genome-wide differential expression analysis of *C. cinerea* S1P and VM. (XLSX 3 MB)

Additional file 2: Table S2: Genes up-regulated in S1P and VM. (XLSX 364 KB)

Additional file 3: Figure S1: RNA-seq data validation by qRT-PCR. The expression of four selected genes was validated by qRT-PCR. A comparable relative expression pattern was found for all the genes evaluated in S1P and VM with both techniques. RNA-seq data corresponds to the mean log2(S1P/VM) of two biological replicates. qRT-PCR data show the mean log2(S1P/VM) of three technical replicates from a single biological replicate of S1P and VM. Bars correspond to standard deviations. Dashed lines indicate the differential gene expression thresholds selected for this study (log2(S1P/VM) = +/-3). (TIFF 413 KB)

Additional file 4: Table S3: RNA-seq expression of reference housekeeping loci. (DOCX 14 KB)

Additional file 5: Table S4: DAVID functional annotation enrichment of genes up-regulated in S1P and VM. (XLSX 54 KB)

Additional file 6: Figure S2: Scheme of fruiting body development in *C. cinerea*. Gene mutations preventing fruiting body development at different stages in *C. cinerea* are shown in blue. White and black sections indicate light and dark periods, respectively, corresponding to 12 h each. Numbers indicate the measured fold (S1P/VM). A mutation in the S1P-specific locus *cfs1* stops sexual development at the initials stage. Figure adapted from Kües U, 2000. (TIFF 524 KB)

Additional file 7: Figure S3: Differential transcription comparison between genes coding for Velvet domain-containing proteins from three different basidiomycetes. Amino acid sequences corresponding to the full set of Velvet domain-containing proteins encoded in the genomes of *C. cinerea*, *L. bicolor* and *S. commune* were retrieved from Pfam (PF11754). SMART domain architecture diagrams are shown in (A). (B) Loci expression in S1P (*C. cinerea* and *S. commune*) or YFB (*L. bicolor*) relative to VM for all the velvet domain-containing proteins in the three basidiomycetes compared. N/E: No expression detected. (TIFF 473 KB)

Additional file 8: Table S5: Orthology and expression of *C. cinerea*, *S. commune* y *L. bicolor* genes. (XLSX 83 KB)

Additional file 9: **Multiple sequence alignment between Velvet-interacting proteins of** ***C. cinerea***, ***L. bicolor***, ***S. commune***, ***A. clavatus***, ***A. fumigatus***, ***A. oryzae***, ***A. niger*** **and** ***A. nidulans***. (PDF 2 MB)

Additional file 10: Figure S4: Differential transcription comparison between genes coding for Velvet-associated proteins from three different basidiomycetes. *C. cinerea*, *L. bicolor* and *S. commune* loci homologous to those encoding VelvetA-associated proteins in the ascomycete *Aspergillus clavatus* (shown on top) were identified by PSI-BLAST (Best hit showing an E-value < 0.005). Differential expression in S1P or YFB relative to VM is shown for the orthologs identified. log2(S1P or YFB/VM) > 0 indicates increased expression in S1P (*C. cinerea* or *S. commune*) or YFB (*L. bicolor*). On the contrary, a log2(S1P or YFB/VM) < 0 represents a decreased expression in S1P or YFB. Blue and white locus IDs differentiate neighboring groups of orthologs in the chart. Expression of *velB* and *kapA* is conserved during sexual development among basidiomycetes. N/E: No expression detected. (TIFF 347 KB)

Additional file 11: Figure S5: Differential transcription comparison between genes coding for transcription factors involved in sexual development in *S. commune* from three different basidiomycetes. Orthologs of transcription factors involved in sexual development in *S. commune* were identified by PSI-BLAST in the genomes of *C. cinerea* and *L. bicolor*. log2((S1P or YFB)/VM) < 0: Down-regulation in S1P or YFB; log2((S1P or YFB)/VM) > 0: up-regulation in S1P or YFB. Domain architecture for each ortholog in the three species compared is shown in the right hand panel. (TIFF 797 KB)

Additional file 12: Table S6: LC-MS analysis of *C. cinerea* S1P and VM. (XLSX 53 KB)

Additional file 13: Table S7: Genes up-regulated in S1P and VM detected by LC-MS. (XLSX 19 KB)

Additional file 14: Table S8: qRT-PCR validation primers. (DOCX 13 KB)
