# Supplementary figures and images for: Comparative transcriptomics of the model mushroom Coprinopsis cinerea reveals tissue-specific armories and a conserved circuitry for sexual development
Source: BMC Genomics. 2014 Jun 19;15(1):492. doi: 10.1186/1471-2164-15-492 (PMC4082614; doi:10.1186/1471-2164-15-492)

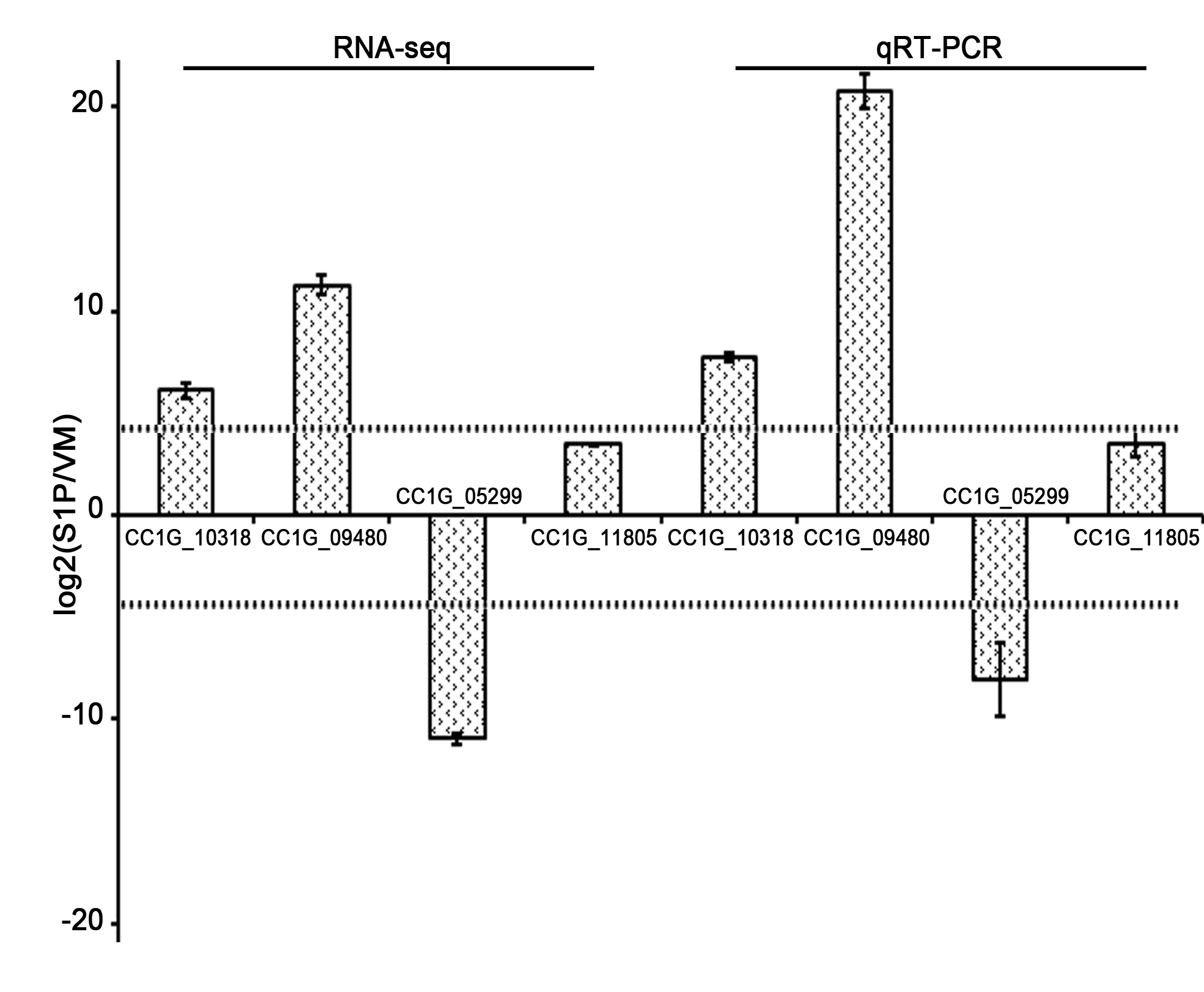

Supplement: Supplementary file 3 — Additional file 3: Figure S1: RNA-seq data validation by qRT-PCR. The expression of four selected genes was validated by qRT-PCR. A comparable relative expression pattern was found for all the genes evaluated in S1P and VM with both techniques. RNA-seq data corresponds to the mean log2(S1P/VM) of two biological replicates. qRT-PCR data show the mean log2(S1P/VM) of three technical replicates from a single biological replicate of S1P and VM. Bars correspond to standard deviations. Dashed lines indicate the differential gene expression thresholds selected for this study (log2(S1P/VM) = +/-3). (TIFF 413 KB) [file 12864_2014_6189_MOESM3_ESM.tiff]

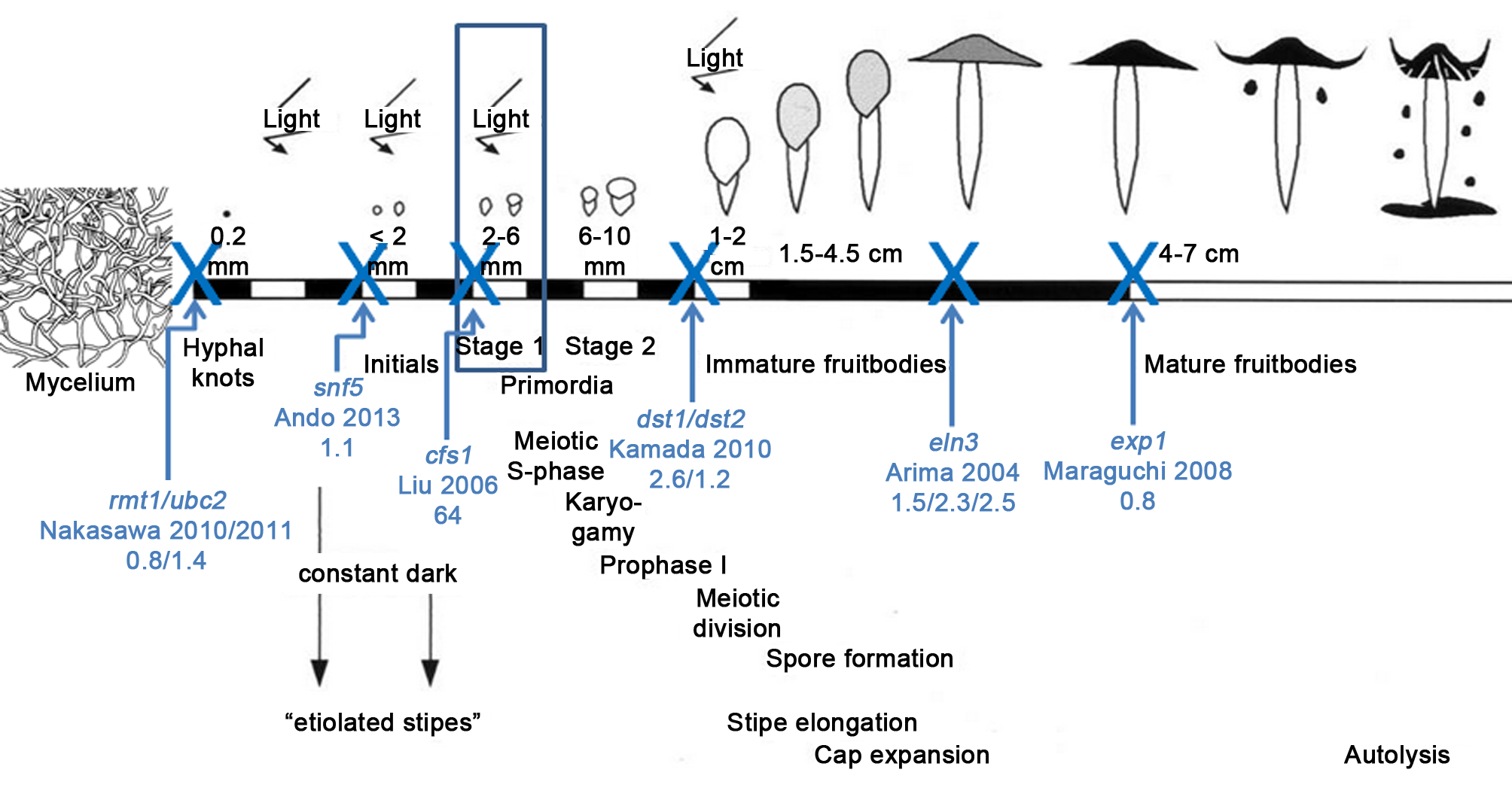

Supplement: Supplementary file 6 — Additional file 6: Figure S2: Scheme of fruiting body development in C. cinerea. Gene mutations preventing fruiting body development at different stages in C. cinerea are shown in blue. White and black sections indicate light and dark periods, respectively, corresponding to 12 h each. Numbers indicate the measured fold (S1P/VM). A mutation in the S1P-specific locus cfs1 stops sexual development at the initials stage. Figure adapted from Kües U, 2000. (TIFF 524 KB) [file 12864_2014_6189_MOESM6_ESM.tiff]

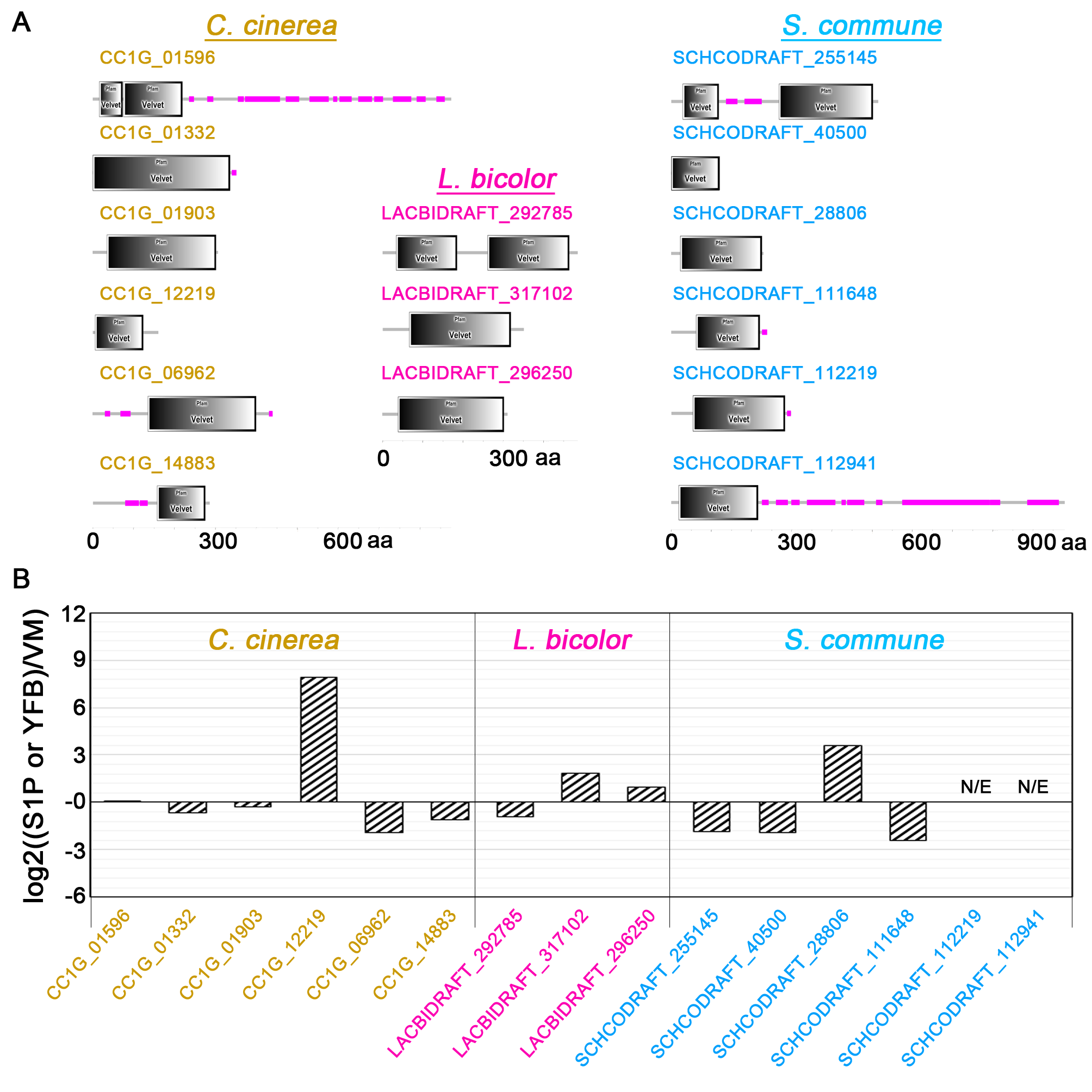

Supplement: Supplementary file 7 — Additional file 7: Figure S3: Differential transcription comparison between genes coding for Velvet domain-containing proteins from three different basidiomycetes. Amino acid sequences corresponding to the full set of Velvet domain-containing proteins encoded in the genomes of C. cinerea, L. bicolor and S. commune were retrieved from Pfam (PF11754). SMART domain architecture diagrams are shown in (A). (B) Loci expression in S1P (C. cinerea and S. commune) or YFB (L. bicolor) relative to VM for all the velvet domain-containing proteins in the three basidiomycetes compared. N/E: No expression detected. (TIFF 473 KB) [file 12864_2014_6189_MOESM7_ESM.tiff]

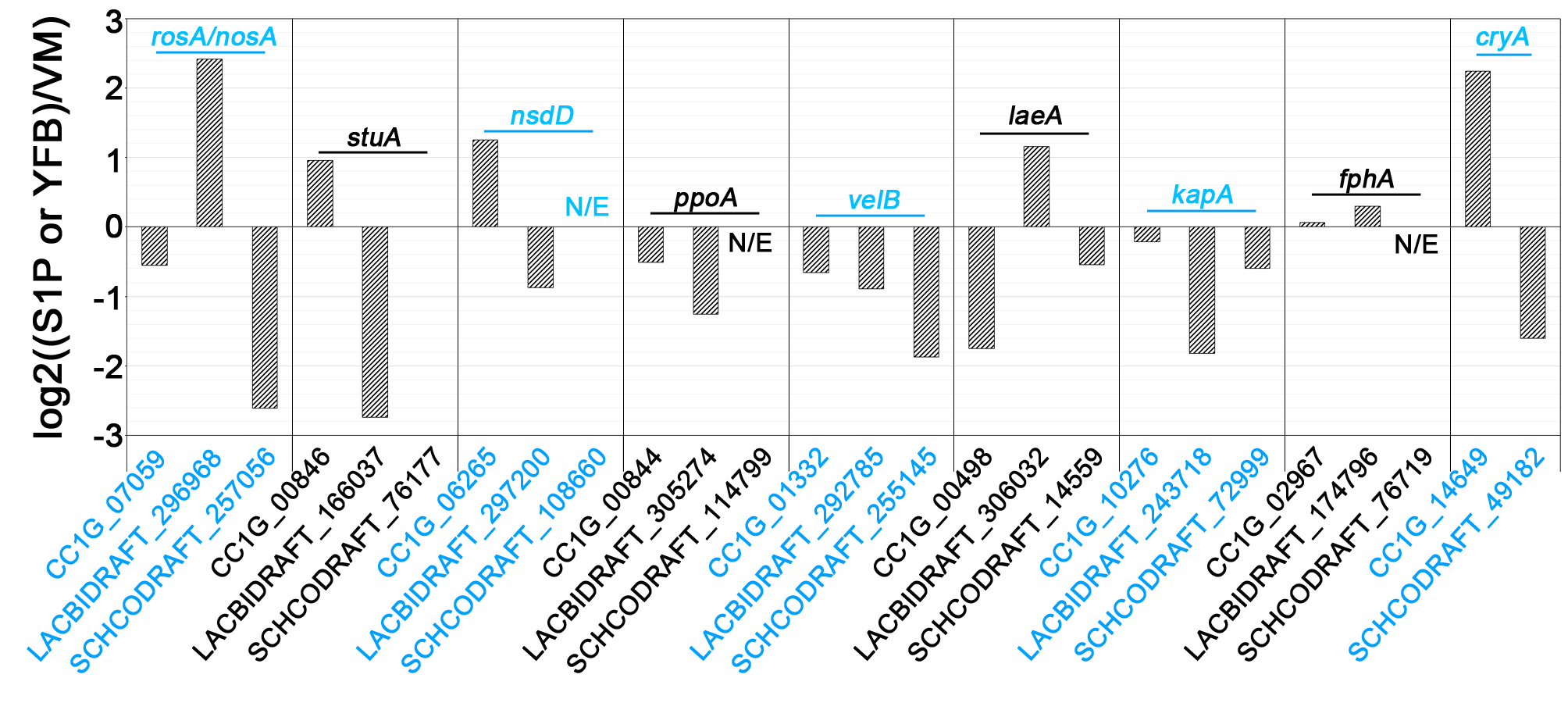

Supplement: Supplementary file 10 — Additional file 10: Figure S4: Differential transcription comparison between genes coding for Velvet-associated proteins from three different basidiomycetes. C. cinerea, L. bicolor and S. commune loci homologous to those encoding VelvetA-associated proteins in the ascomycete Aspergillus clavatus (shown on top) were identified by PSI-BLAST (Best hit showing an E-value < 0.005). Differential expression in S1P or YFB relative to VM is shown for the orthologs identified. log2(S1P or YFB/VM) > 0 indicates increased expression in S1P (C. cinerea or S. commune) or YFB (L. bicolor). On the contrary, a log2(S1P or YFB/VM) < 0 represents a decreased expression in S1P or YFB. Blue and white locus IDs differentiate neighboring groups of orthologs in the chart. Expression of velB and kapA is conserved during sexual development among basidiomycetes. N/E: No expression detected. (TIFF 347 KB) [file 12864_2014_6189_MOESM10_ESM.tiff]

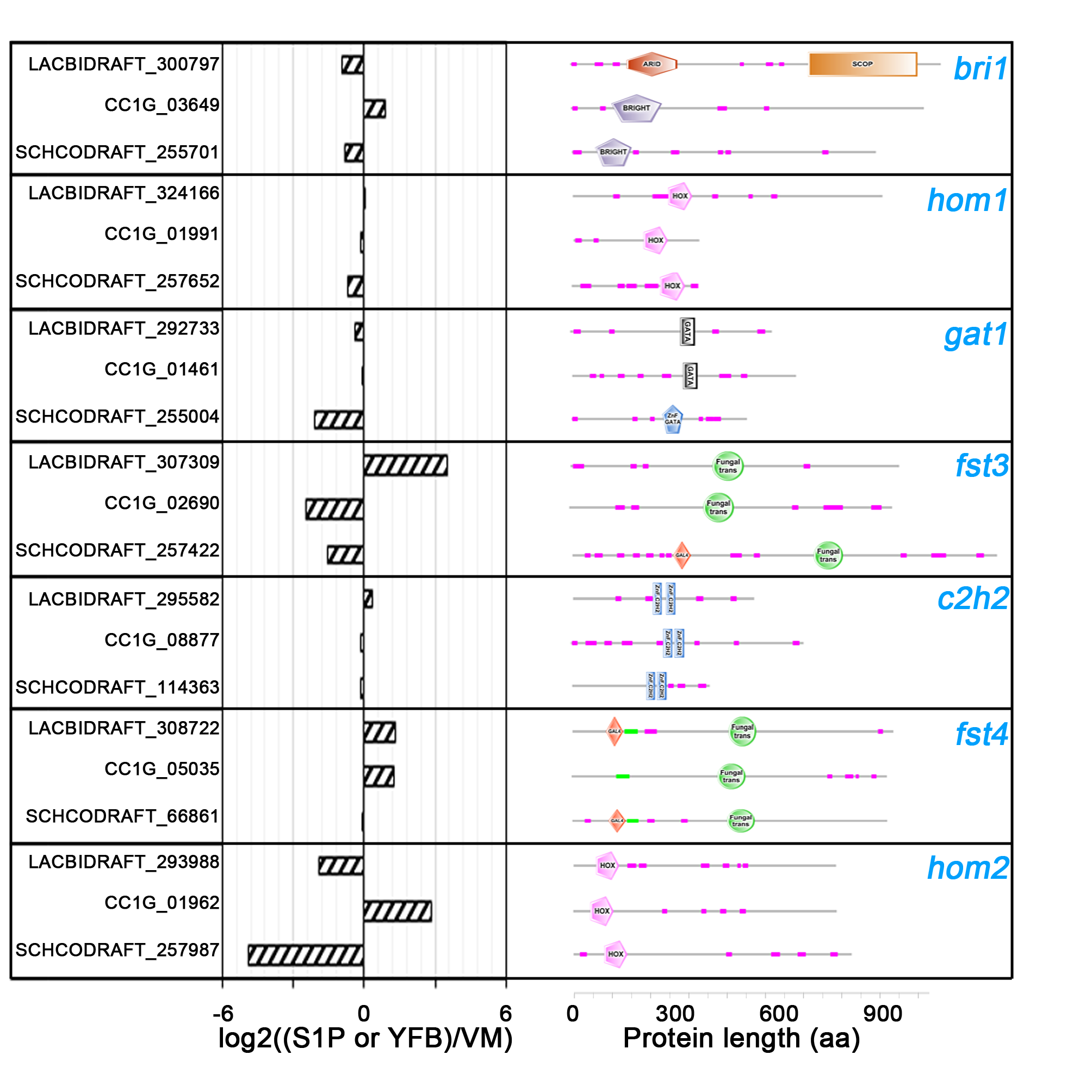

Supplement: Supplementary file 11 — Additional file 11: Figure S5: Differential transcription comparison between genes coding for transcription factors involved in sexual development in S. commune from three different basidiomycetes. Orthologs of transcription factors involved in sexual development in S. commune were identified by PSI-BLAST in the genomes of C. cinerea and L. bicolor. log2((S1P or YFB)/VM) < 0: Down-regulation in S1P or YFB; log2((S1P or YFB)/VM) > 0: up-regulation in S1P or YFB. Domain architecture for each ortholog in the three species compared is shown in the right hand panel. (TIFF 797 KB) [file 12864_2014_6189_MOESM11_ESM.tiff]
