# Supplementary material for: Comparative transcriptomics of the model mushroom Coprinopsis cinerea reveals tissue-specific armories and a conserved circuitry for sexual development
Source: BMC Genomics. 2014 Jun 19;15(1):492. doi: 10.1186/1471-2164-15-492 (PMC4082614; doi:10.1186/1471-2164-15-492)
Supplement: Supplementary file 4 — Additional file 4: Table S3: RNA-seq expression of reference housekeeping loci. (DOCX 14 KB) [file 12864_2014_6189_MOESM4_ESM.docx]

|  |  | **Reads/kb** | | | |  |  |
| --- | --- | --- | --- | --- | --- | --- | --- |
| **Locus** | **Functional annotation** | **VM1** | **VM2** | **S1P1** | **S1P2** | **log2(S1P/VM)** | **Reference** |
| CC1G_06184 | Beta tubulin | 3187 | 4296 | 2746 | 3543 | **-0.2510** | Wan H 2011 |
| CC1G_04743 | Tubulin beta chain | 57352 | 69507 | 140002 | 168645 | **1.2827** | Wan H 2011 |
| CC1G_07639 | Histone H2B | 49428 | 50071 | 144222 | 173258 | **1.6739** | Ferreira 2012 |
| CC1G_03523 | Histone H2B | 44912 | 38795 | 77045 | 79233 | **0.9007** | Ferreira 2012 |
| CC1G_13048 | Actin | 677 | 624 | 792 | 878 | **0.3605** | Huggett 2005 |
| CC1G_08232 | Actin | 96495 | 116431 | 112483 | 108304 | **0.0523** | Huggett 2005 |
| CC1G_09116 | GADPH | 119239 | 151363 | 40820 | 40636 | **-1.7321** | Huggett 2005 |
| CC1G_09117 | GADPH | 205 | 187 | 119 | 121 | **-0.7087** | Huggett 2005 |
| CC1G_11833 | Ubiquitin | 99610 | 150276 | 185548 | 200736 | **0.6284** | Silveira 2009 |
| CC1G_00876 | Ubiquitin | 80981 | 91185 | 127562 | 128692 | **0.5738** | Silveira 2009 |
| CC1G_03676 | Ubiquitin C | 353488 | 272284 | 149754 | 176153 | **-0.9412** | Silveira 2009 |
| CC1G_09572 | Cyclophilin | 333724 | 296676 | 103503 | 92140 | **-1.6880** | Langnaese 2008 |
| CC1G_15352 | Ribosomal protein S27a | 255 | 232 | 141 | 150 | **-0.7416** | de Oliveira 2011 |
| CC1G_04355 | Ribosomal protein L19 | 80930 | 74132 | 55940 | 58059 | **-0.4438** | de Oliveira 2011 |
| CC1G_00758 | Ribosomal protein L11 | 58743 | 49275 | 64729 | 61966 | **0.2301** | de Oliveira 2011 |
| CC1G_13649 | Ribosomal protein L32 | 83383 | 81823 | 75132 | 78869 | **-0.1013** | de Oliveira 2011 |
| CC1G_03927 | Hsp90 | 16541 | 22271 | 22405 | 31048 | **0.4618** | Aursnes 2011 |

**Table S3.** Reference housekeeping loci are constitutively expressed in *C. cinerea* A43mutB43mut.
