# Supplementary material for: Comparative transcriptomics of the model mushroom Coprinopsis cinerea reveals tissue-specific armories and a conserved circuitry for sexual development
Source: BMC Genomics. 2014 Jun 19;15(1):492. doi: 10.1186/1471-2164-15-492 (PMC4082614; doi:10.1186/1471-2164-15-492)
Supplement: Supplementary file 9 — Additional file 9: Multiple sequence alignment between Velvet-interacting proteins of C. cinerea, L. bicolor, S. commune, A. clavatus, A. fumigatus, A. oryzae, A. niger and A. nidulans. (PDF 2 MB) [file 12864_2014_6189_MOESM9_ESM.pdf]

ClustalW multiple sequence alignment Velvet-associated proteins

Acla: *A. clavatus*
Afum: *A. fumigatus*
Aory: *A. oryzae*
Anig: *A. niger*
Anid: *A. nidulans*
LACBIDRAFT: *L. bicolor*
SCHCODRAFT: *S. commune*
CC1G: *C. cinerea*

* Identical
: Strongly similar
. Weakly similar

RosA/NosA

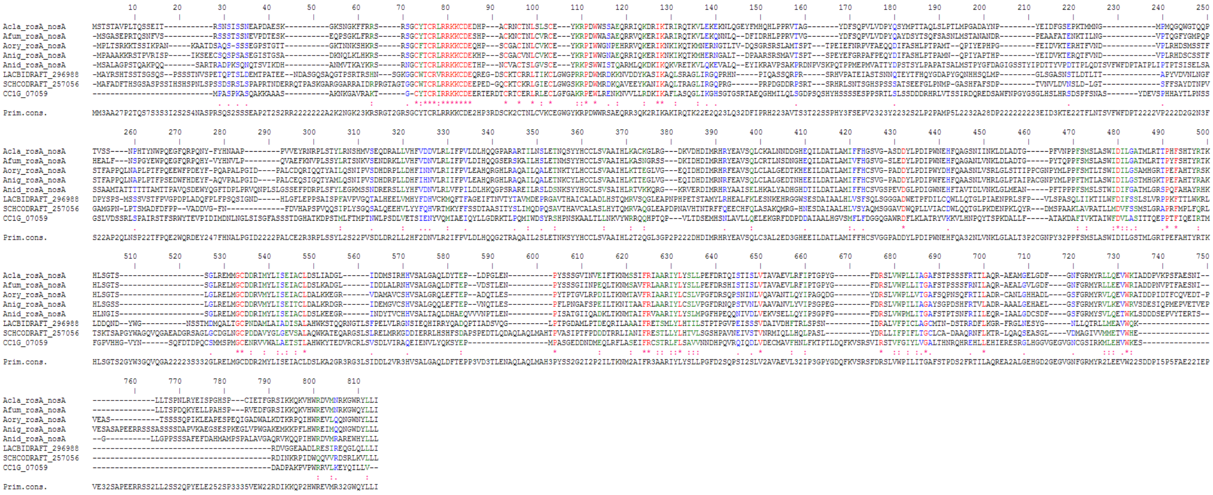

StuA

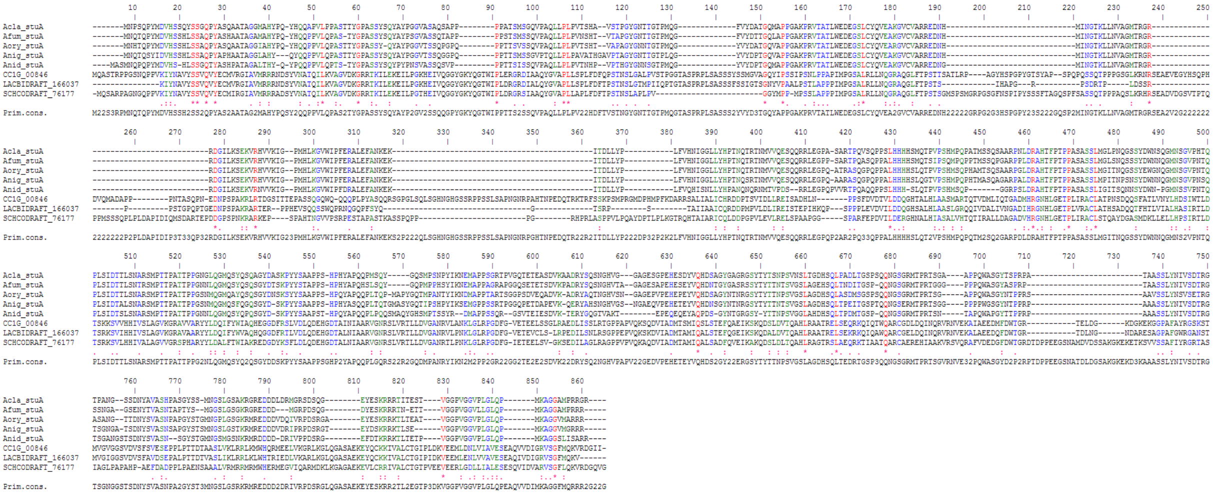

PpoA

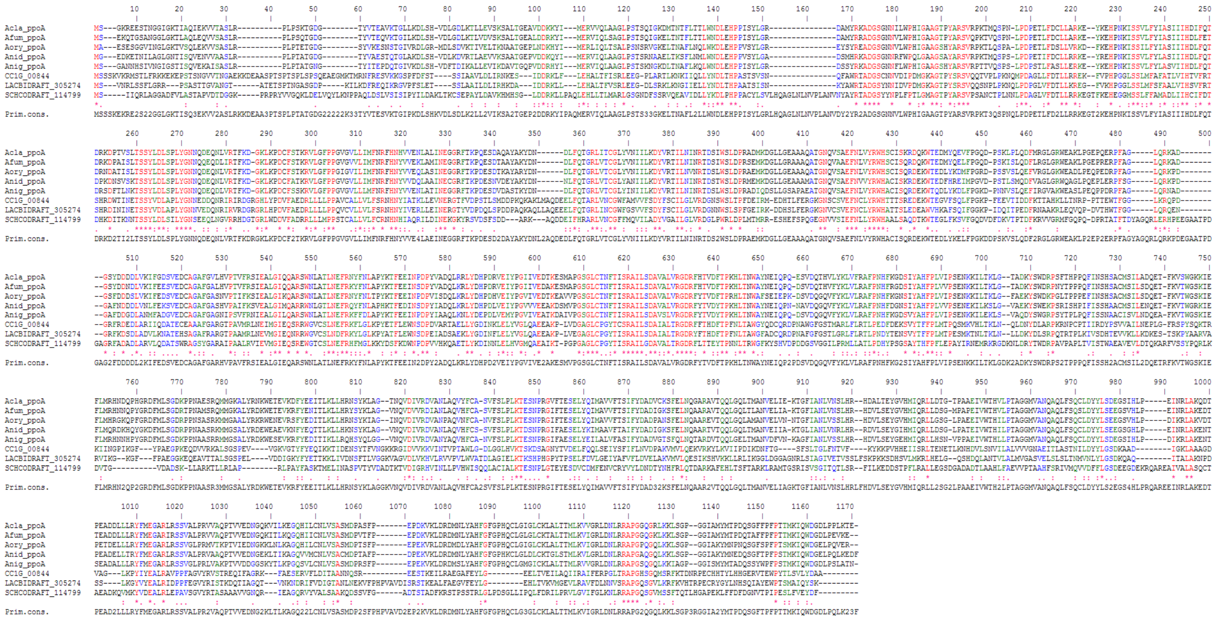

VelB

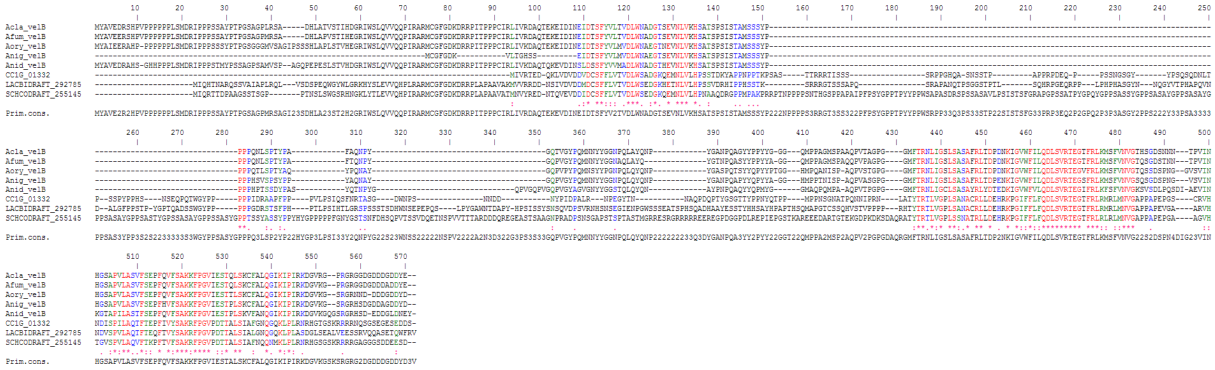

NsdD

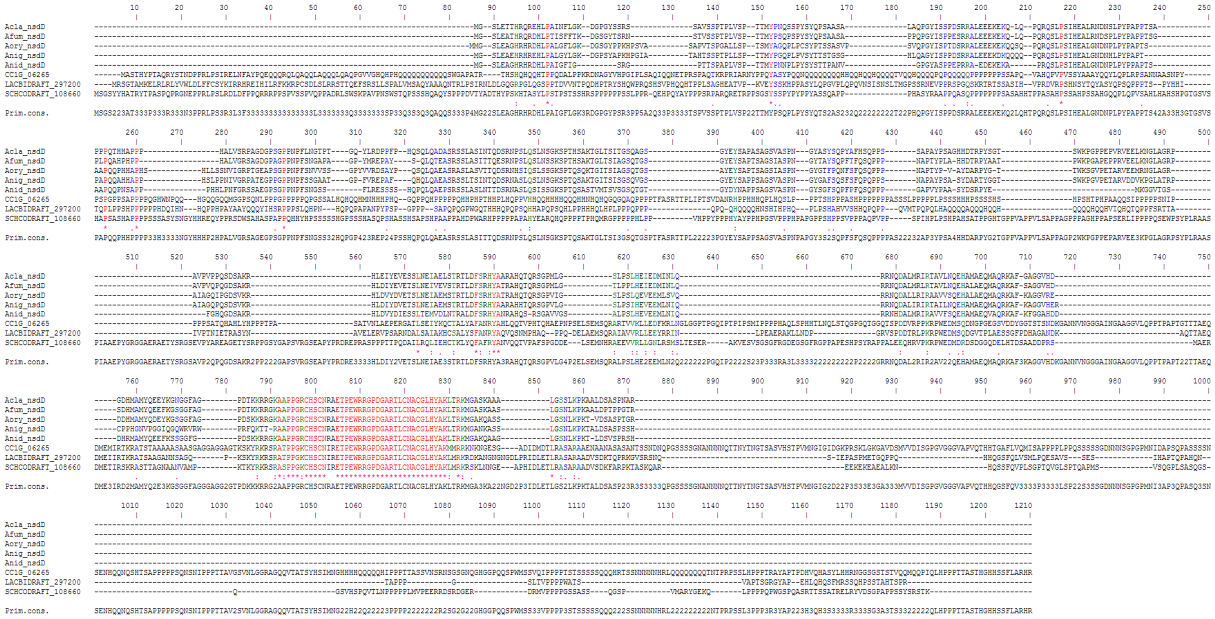

LaeA

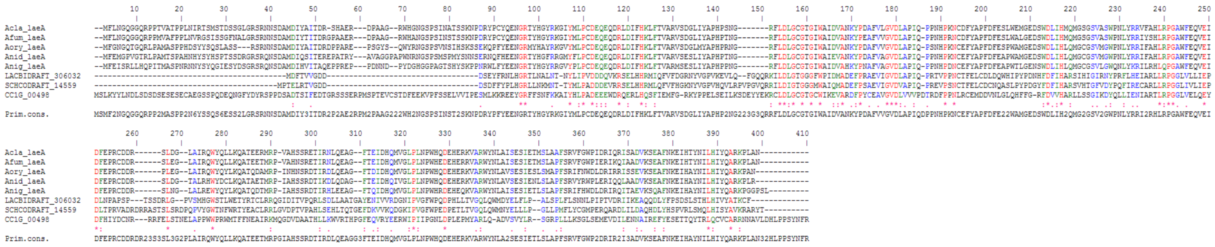

KapA

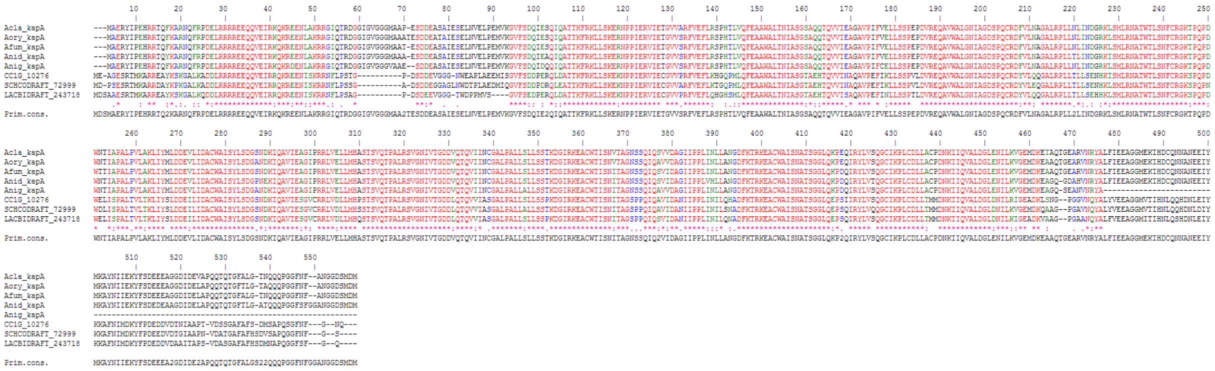

FphA

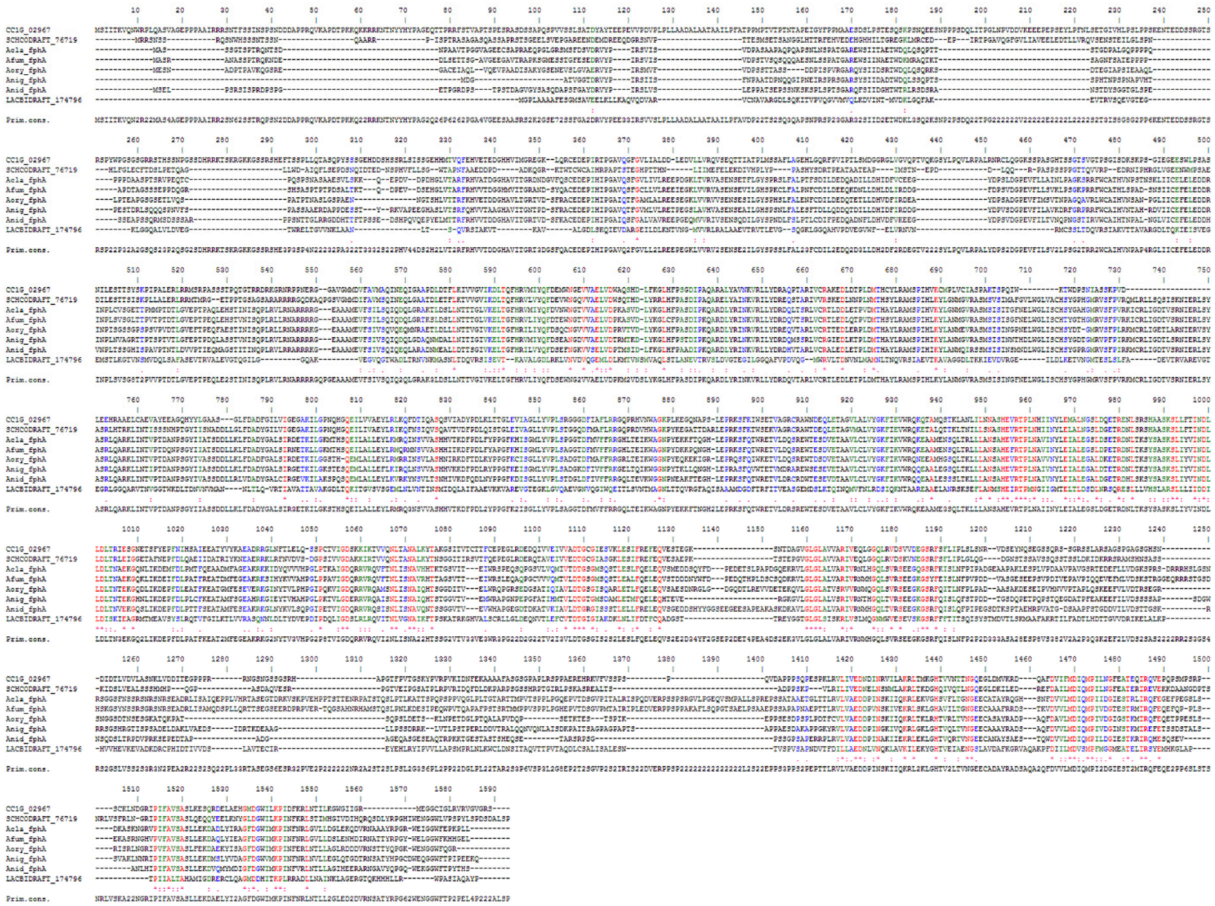

CryA

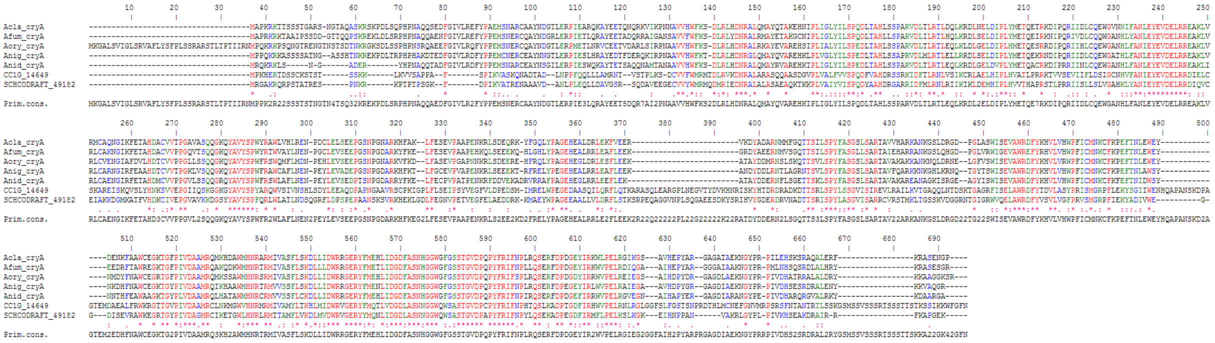
Acla_cryA
Afum_cryA
Aory_cryA
Anig_cryA
Anid_cryA
CC1G_14649
SCHCODRAFT_49182
Prim.cons.
