# Supplementary material for: Comparative transcriptomics of the model mushroom Coprinopsis cinerea reveals tissue-specific armories and a conserved circuitry for sexual development
Source: BMC Genomics. 2014 Jun 19;15(1):492. doi: 10.1186/1471-2164-15-492 (PMC4082614; doi:10.1186/1471-2164-15-492)
Supplement: Supplementary file 14 — Additional file 14: Table S8: qRT-PCR validation primers. (DOCX 13 KB) [file 12864_2014_6189_MOESM14_ESM.docx]

| **Locus** | **Functional annotation** | **Primer name** | **Sequence (5'→3')** | **Tm°C** |
| --- | --- | --- | --- | --- |
| CC1G_04743 | Tubulin beta chain | TubM1Fw | GTCATGTCCGGTATCACCAC | 62 |
| CC1G_04743 | Tubulin beta chain | TubM1Rv | GGGAAAGGAACCATGTGGA | 61 |
| CC1G_05299 | Ricin B-fold protein | CC1G_05299FqRTPCR | CGGCGACAACCAGCTTTGGGACTTTG | 72 |
| CC1G_05299 | Ricin B-fold protein | CC1G_05299RqRTPCR | GACACCGATCCTCCAGACGGATC | 68 |
| CC1G_09480 | Cospin | PICFwqRTPCR | ACGTCTTCACCGTCGTGAATGC | 67 |
| CC1G_09480 | Cospin | PICRvqRTPCR | TCGACTTGGGTGAAGGTAAAGAGC | 66 |
| CC1G_10318 | Pore-forming protein | CaerofqRTPCR | TTTGAGCTCGGGCAGAGGTTAACG | 68 |
| CC1G_10318 | Pore-forming protein | CaeroRqRTPCR | AAGCGTGGCCCTCTCCGTGTAAG | 70 |
| CC1G_11805 | Pore-forming protein | CC11805RTPCRFw | GGCATCGAACTCGGACAAACGTTCACTTTC | 71 |
| CC1G_11805 | Pore-forming protein | CC11805RTPCRRv | AGTTGGTGATCCTCTCCGTGTAAG | 65 |

**Table S8.** qRT-PCR primers used during RNA-seq validation.
